# Multiscale Perspectives on Solid-Phase Astrochemistry: Laboratory, Computation, and Open Questions

**DOI:** 10.1007/s11214-025-01228-9

**Published:** 2025-11-05

**Authors:** Matthew D. Dickers, Duncan V. Mifsud, Nigel J. Mason, Felipe Fantuzzi

**Affiliations:** 1 Physics and Astronomy, School of Engineering, Mathematics and Physics, University of Kent, Canterbury, CT2 7NH UK; 2 HUN-REN Institute for Nuclear Research (ATOMKI), Debrecen, H-4026 Hungary; 3 Chemistry and Forensic Science, School of Natural Sciences, University of Kent, Canterbury, CT2 7NH UK

**Keywords:** Astrochemistry, Laboratory methods, Computational simulations, Ice films, Multiscale modelling

## Abstract

This review provides an outline of the key processes behind the formation of dust grains in the interstellar medium, the growth of thin ice mantles upon their surface, and their impact on the chemistry that can take place at the centre of cold molecular clouds. These dust grains provide a vital surface to catalyse complex chemistry, without which many of the complex molecules now observed in the interstellar medium could not form. We highlight the experimental methodology by which ice analogues may be grown and analysed in a laboratory setting, as well as their shortcomings; in particular, the limitations on experimental deposition timescales that present a particular problem when compared to the accretion rates in the interstellar medium. Potential solutions to these constraints are underscored through computational simulations, with particular emphasis on the impact that newly emerging multiscale methods may have on future models of ice mantle formation.

## Introduction

To date, around 338^1^ different molecular species in both the solid and gas phases (Endres et al. 2016; McGuire 2022) have been observed in the interstellar medium (ISM), and with the commencement of *James Webb Space Telescope* (JWST) operations in 2022 we can only expect this number to rise in the coming years (McClure et al. 2023). Many of these observed molecules cannot be easily formed in the gas phase, and thus surface chemistry and the processing of ice mantles adsorbed on dust grains have been put forward as pivotal mechanisms by which several molecules, including complex organic molecules (COMs), can be formed in the ISM in the abundances in which they are observed (Tielens and Hagen 1982; Herbst and Dishoeck 2009; Potapov and McCoustra 2021).

Ice mantles form from the accretion of gas-phase molecules onto the surfaces of cold silicate or carbonaceous dust grains in dense interstellar clouds, where temperatures can reach as low as 10 K (Muñoz Caro and Escribano 2018; Öberg 2016). These mantles can form multiple layers, and typically consist of CO, N_2_, CO_2_, NH_3_, CH_4_, and CH_3_OH, among others, with H_2_O being the most common constituent, making up over 60% of the total ice observed (Whittet 2022). The structure of these ice mantles is highly dependent on the temperature of the dust grain, and the rate at which molecules are accreted (Materese et al. 2021; Muñoz Caro and Escribano 2018).

Processing of ice mantles takes place through various means, including heating, shocks from nearby supernovae events, and UV and cosmic ray irradiation, leading to photochemistry and radiation chemistry (Öberg 2016; Arumainayagam et al. 2019). It has also been demonstrated that the chemistry that takes place is dependent on the phase (crystalline or amorphous) of these ice mantles (Orlando and Sieger 2003; Grieves and Orlando 2005; Mason et al. 2006; Zheng et al. 2007; Mifsud et al. 2022a,b,c). The products of this chemistry create a rich and diverse range of molecules present in the ISM, many of which are precursors to organic molecules, or COMs themselves. As such, icy dust grains may represent the starting point to the formation of life-relevant complex organics in the ISM.

This review summarises the key details and processes that lead to the formation of ice mantles on interstellar dust grains, discussing the role of both experimental and computational studies in revealing the mechanisms behind these formation processes. It also provides an overview and critical analysis of major techniques used in these studies. The motivation behind this review is to establish the reliability of current models of ice mantle formation in the context of the conditions in which these icy grains form in the ISM. Conditions analogous to the ISM are difficult to reproduce in the laboratory; thus, we review the results of experimental studies and evaluate how these may compare to the formation of interstellar ice mantles under true ISM conditions.

This review is presented in seven parts; we first provide an overview of dust in the ISM in Sect. 2, including the types of dust present, grain structures, and the dust life cycle. We then examine the formation of ice mantles in the ISM in Sect. 3, covering the types of ice present, the processes behind mantle formation, and the observed ice morphologies. In Sect. 4, we provide an overview of the different methods of ice mantle processing and the resulting reaction products. We present the state of the art in both experimental and computational approaches in Sects. 5 and 6, using illustrative examples to highlight the challenges faced by the community. These topics have been previously discussed in other reviews (Öberg 2016; Arumainayagam et al. 2019; Sandford et al. 2020; Cuppen et al. 2024), albeit often in isolation, whereas here we aim to provide a more comprehensive interlinked review. Nevertheless, for a reader who feels familiar with these topics they may skip to Sect. 7. In this final section we introduce a number of case studies that are representative of open questions in the field of laboratory astrochemistry. In particular, these are case studies that may be addressed from a computational perspective. They include the accuracy of current experimental studies in the context of replicating the slow ice mantle growth conditions of the ISM, modelling of irradiation-induced chemistry in ice mantles, and the formation and growth of dust grains.

## Interstellar Dust

Initially identified in 1912 by Slipher (1912) from spectral observations of the Pleiades, the presence of “pulverulent matter” was further inferred through observations of stellar extinction in 1930 by Trumpler (1930). While making up only 1% of the ISM by mass (the remaining 99% being gas), interstellar dust plays a key role in ISM processes. Dust is fundamental to the thermal properties of the ISM; $\sim 30\%$ of all starlight is absorbed and re-emitted as lower wavelength infrared (IR) radiation (Sellgren 1984; Dorschner 2001; Bernstein et al. 2002a), cooling the surrounding gas in molecular clouds to allow star formation to take place (Asano et al. 2013). The dust also shields molecular clouds from the harsh ultraviolet (UV) radiation emitted by massive stars, which would otherwise destroy existing molecules (Muñoz Caro and Escribano 2018). This protection allows molecules to survive longer within these environments, leading to complex gas-phase, surface, and radiation chemistry. Such chemistry is further aided by later star formation within clouds, as detailed in Sect. 4.

Interstellar dust starts its life in the atmospheres of asymptotic giant branch (AGB) stars. The mass of these stars (1 M_⊙_-4 M_⊙_) are too low to undergo supernovae. Thus, as they reach the end of their lives, they begin to expand and lose the majority of their mass due to radiation pressure (Höfner and Olofsson 2018). AGB stars with oxygen-rich atmospheres will largely produce silicate dust (Waters et al. 1996; Molster et al. 1999; Gail et al. 2009; Gobrecht et al. 2016), while stars with carbon-rich atmospheres will largely produce carbonaceous dust (Cherchneff and Cau 1999; Gail et al. 2009; Schneider et al. 2014; Nanni et al. 2018, 2019, 2021). These types of dust will be explored in more detail in Sects. 2.2 and 2.3, respectively. Other materials such as metals, metallic oxides and sulphides are also produced through these processes; however, in much lower abundances (Dorschner and Henning 1995). More exotic elements heavier than iron form exclusively in supernova explosions, and thus make up a very small fraction of interstellar dust (Jones and Nuth 2011). Interstellar dust grains primarily originate from outflows of AGB stars and supernovae (Matsuura et al. 2011; Sarangi et al. 2018; Micelotta et al. 2018), with recent studies suggesting that $\sim 50\%$ of silicate grains form from AGB stars, and $>30\%$ from supernovae (Hoppe et al. 2021, 2022). These grains typically range in size from 200 Å to 0.1 μm (Gobrecht et al. 2016; Nanni et al. 2021).

Supernovae may play a key role in the life cycle of interstellar dust, as shocks from these stellar explosions can rapidly destroy ISM dust (Jones et al. 1994, 1996). However, the presence of dust in the ISM indicates that mechanisms must exist to either protect or replenish this destroyed material. Jones and Nuth (2011) posit that dust inside denser clumps is shielded from these shocks. It was noted in 1979 by Draine and Salpeter (1979) that there was a discrepancy between the silicate dust observed in the ISM and dust estimated to have originated from stars. As such, it seems that supernovae do indeed lead to significant dust destruction, with models estimating that as little as 10% of interstellar dust survives these shocks (Draine 2003; Zhukovska et al. 2008; Draine 2009). Instead, the majority of the total interstellar dust in the ISM appears to form in the centres of molecular clouds, where temperatures can reach as low as 10-20 K. At these low temperatures, small dust grain fragments from supernova shocks condense to form larger grains (Draine 2009; Rouillé et al. 2014; Krasnokutski et al. 2014; Fulvio et al. 2017). Through this cold condensation, grains can reach micrometres in size (Draine 2003).

### Life Cycle of Dust

**Fig. 1:**
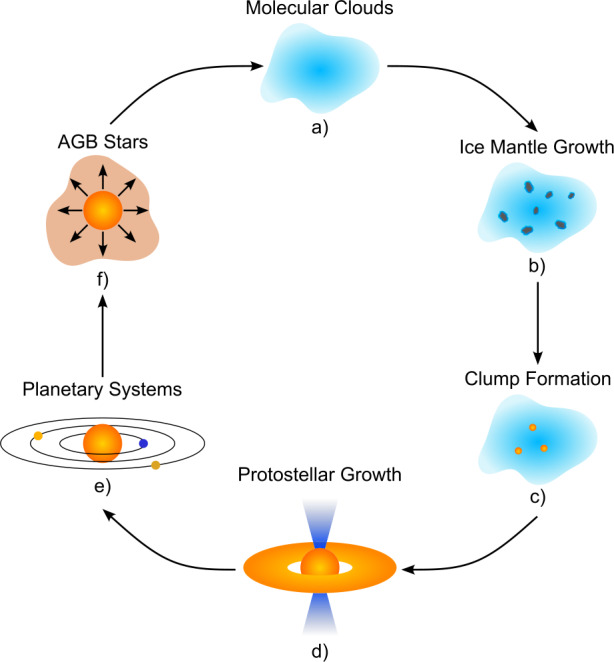
Schematic illustration of the life cycle of interstellar dust. Dust accumulates in interstellar clouds (**a**), where they may act as the surface on which ice mantles may subsequently form (**b**). The collapse of the cloud into star-forming cores (**c**) eventually leads to the development of protostars (**d**), which then evolve into planetary systems (**e**). At the end of an AGB star’s life cycle, new dust grains can be formed in the stellar atmosphere and ejected into the ISM by stellar winds (**f**)

The overall life cycle of interstellar dust was outlined by Tielens (1998); dust is initially formed in the atmospheres of AGB stars, before being distributed throughout the ISM by outflows and supernovae. Dust that survives these processes eventually coalesces into molecular clouds, in the interiors of which ice mantles form and the dust cools. This cold dust is able to collapse under its own gravity to form protostars, and eventually stellar and planetary systems. This cycle then repeats, with dust once again being formed in the atmospheres of these stars when they approach the end of their lives. Figure 1 shows an illustration of this life cycle.

### Silicate Grains

Silicate grains are arguably the most abundant component of cosmic dust, dominating environments from the diffuse ISM to protoplanetary discs and distant quasars (Henning 2010; Dwek et al. 2014). Their presence is revealed by characteristic IR bands; most notably, a strong absorption feature around 10 μm arising from the Si–O stretching mode and another near 18 μm produced by the O–Si–O bending mode (McCarthy et al. 1980). Typically composed of iron- and magnesium-bearing olivine (Mg_2*x*_Fe_2−2*x*_SiO_4_) and pyroxene (Mg_*x*_Fe_1−*x*_SiO_3_) types (Muñoz Caro and Escribano 2018), interstellar silicates are predominantly amorphous—with approximately 95% of the silicate mass in the diffuse ISM in an amorphous state (Li and Draine 2002; Dorschner and Henning 1995; Draine 2003; Henning 2010). The disordered network of SiO_4_ tetrahedra in these amorphous grains gives rise to broad, smooth IR features.

In contrast, crystalline silicates produce numerous sharp, well-defined spectral features spanning the mid- to far-IR. Laboratory measurements of minerals such as forsterite (Mg_2_SiO_4_) and enstatite (MgSiO_3_) reveal distinct emission bands at approximately 9.4, 10, 11.3, 16, 19–23, 27–28, and 33–35 μm, with the precise positions and intensities depending on both chemical composition and grain shape (Henning 2010). Crystalline grains are thought to form in the warm circumstellar regions near stars, where heating transforms amorphous SiO_4_ networks into well-ordered lattices (Gail 2004). However, once these grains are injected into the diffuse ISM, destructive processes (such as shocks, sputtering, and ion bombardment) efficiently amorphise them, which accounts for their relative scarcity (typically only a few per cent of the total silicate mass) in such environments (Bringa et al. 2007; Henning 2010).

Within our Solar System, silicates are found not only on planetary surfaces and in asteroids and comets but also in interplanetary dust particles and in the interstellar dust stream passing through the heliosphere (Sterken et al. 2019). Cometary dust, for instance, often displays distinct crystalline silicate signatures, as seen in Hale–Bopp, implying that at least part of the dust underwent high-temperature restructuring in the inner solar nebula before being transported outward (Crovisier et al. 1997; Henning 2010). Similarly, asteroidal silicates reveal both amorphous and crystalline phases in ground-based spectroscopy (Feierberg et al. 1983; Emery et al. 2006), while primitive meteorites may preserve presolar silicate grains with exotic isotopic signatures that trace their origins to asymptotic giant branch (AGB) star outflows or supernova remnants.

Young stellar objects (YSOs) also exhibit prominent mid-IR silicate features that appear in either absorption or emission, depending on their dust geometry and evolutionary stage. YSOs are typically classified from Class 0 to Class III based on the shape of their spectral energy distributions: Class 0/I sources are deeply embedded protostars enshrouded by dense envelopes of gas and dust; Class II objects, such as T Tauri and Herbig Ae/Be stars, host substantial protoplanetary discs; and Class III systems display only residual, debris-like discs. In many T Tauri and Herbig Ae/Be stars, the disc’s upper layers can attain temperatures of several hundred kelvin, which gives rise to strong silicate emission near 10 μm (Natta et al. 2000; Bouwman et al. 2001; Kessler-Silacci et al. 2006). IR observations from the *Infrared Space Observatory* (ISO) and the *Spitzer Space Telescope* (SST) have revealed that these discs often harbour mixtures of amorphous and crystalline silicates: crystalline forsterite and enstatite characterise the warmer, inner regions, while amorphous grains dominate the cooler, outer zones (Watson et al. 2009; Henning 2010). Recent studies even indicate that crystallisation may commence at very early protostellar stages; for instance, Do-Duy et al. (2020) report an 11.1 μm crystalline-forsterite (Mg_2_SiO_4_) absorption band in deeply embedded YSOs. Crystalline forsterite makes up the end member of the olivine solid-solution series.

Shock-induced processes further modify the dust. In NGC 1333 IRAS 4A, for example, Koumpia et al. (2017) demonstrated that powerful outflows disrupt the protostellar envelope, sputtering dust mantles and altering the local chemistry. This picture is bolstered by the wide-band millimetre survey of Quitián-Lara et al. (2024), which identified nearly 1500 transitions from 97 molecular species (including numerous S-bearing and N-bearing molecules) across 72–276 GHz. Their results reveal strong SiO and other shock tracer emissions, confirming that dynamic outflows catalyse significant dust reprocessing.

Evolved stars, particularly AGBs, are major producers of dust in galaxies. In oxygen-rich (M-type) environments where the C/O ratio is less than unity and nearly all carbon is locked in CO, the excess oxygen is available to form refractory compounds. Under high-temperature conditions ($T> {700}$ K) the chemical equilibrium in the outflows favours the condensation of nearly Fe-free crystalline silicates such as forsterite and enstatite (Gail 2010; Henning 2010). At lower temperatures ($T \le {600}$ K) increasing incorporation of Fe results in the formation of amorphous silicates with ‘dirty’ optical properties (Ossenkopf et al. 1992; Dorschner and Henning 1995). Although broad, structureless IR bands near 10 and 18 μm have long been ascribed to amorphous silicates (Draine and Lee 1984; Henning 2010), detailed analyses of spectra from the ISO and SST have revealed diverse band shapes and positions that depend on grain size, morphology and degree of crystallinity (Waters et al. 1996; Jäger et al. 1998; Molster et al. 2002a,b; Yamamura et al. 2000). Traditional IR spectroscopy associates narrow features with crystalline materials and broad features with amorphous ones (Jones and Merrill 1976; Kemper et al. 2004, 2005), yet recent studies (Zamirri et al. 2019) demonstrate that, at nanometre sizes (approximately 1–5 nm), even structurally crystalline grains may exhibit broad features because surface relaxation and disorder mask their intrinsic crystalline ‘fingerprints’ (Li et al. 2007; Vollmer et al. 2009). These broad features also contain substructure from the 10 μm band, in addition to narrow bands not seen in amorphous grains. In oxygen-rich outflows, silicate dust may form either by condensation onto pre-existing aluminium oxide seeds (Salpeter 1977; Ferrarotti and Gail 2006) or by direct nucleation from the gas phase when supersaturation is reached (Gail et al. 2013), and studies of supergiants have introduced non-stoichiometric, iron-bearing silicate models lying between olivine-like and pyroxene-like compositions, with Fe present both as lattice inclusions and as a separate metallic phase (Gail et al. 2020; Tamanai et al. 2017; Keller and Messenger 2011).

Observational studies of oxygen-rich post-AGB stars further constrain the dust formation process and its evolution. For example, Dell’Agli et al. (2023) examined a sample of oxygen-rich post-AGB stars in the Milky Way and found that the optical depth of the dusty envelope and the spatial distribution of the dust are strongly correlated with the progenitor’s mass and metallicity, with more massive, metal-rich stars producing thicker dust shells and faster-expanding outflows. Detailed spectral energy distribution fitting, combined with radiative transfer modelling (using, for example, the DUSTY code; Ivezic et al. 1999) and stellar evolution models (Busso et al. 1999; Herwig 2005; Karakas and Lattanzio 2014), indicates that stars descending from more massive AGB progenitors (typically $\gtrsim {3}$ M_⊙_) which experience hot bottom burning (Blöcker and Schönberner 1991) produce larger quantities of silicate dust. This results in higher IR optical depths and faster-expanding dusty shells (Goldman et al. 2017), while metal-poor stars form little dust owing to the scarcity of silicon and aluminium (Loon 2000; Ferrarotti and Gail 2006). Together with the nanoscale effects that complicate the spectral distinction between crystalline and amorphous grains (Zamirri et al. 2019), these findings imply that refined observational technique, including, for instance, X-ray absorption (Zeegers et al. 2017), and more detailed theoretical models that incorporate both the evolution of stellar outflows and the nanostructure of dust are required to fully characterise the nature and crystalline fraction of interstellar silicate dust.

On galaxy-wide scales, SST surveys have documented the 10- and 18 μm silicate absorption features in various extragalactic environments, including ultraluminous infrared galaxies (ULIRGs) and high-redshift, dust-enshrouded quasars (Houck et al. 2005; Hao et al. 2007; Henning 2010). In some of these extreme systems, crystalline silicate features can be significantly stronger, in a mass-fraction sense, than those in the Milky Way’s diffuse ISM (Spoon et al. 2006). This stark contrast highlights the profound influence that intense star formation or active galactic nucleus (AGN) activity can exert on dust formation, destruction, and transport. Type I AGN, in which the broad-line region is directly visible, sometimes exhibit weak silicate emission at 10 μm or 18 μm when observed at favourable inclinations or in the presence of clumpy dust distributions (Hao et al. 2005; Sturm et al. 2005). However, this emission likely originates from extended, optically thin regions rather than the hot inner walls of a torus.

Recent hydrodynamical simulations have further advanced our understanding of these processes. Dubois et al. (2024) implemented a dust evolution model in the ramses code (Teyssier 2002) that follows two representative grain sizes (5 nm and 0.1 μm) and distinguishes between carbonaceous and silicate compositions. Their isolated disc galaxy simulations, spanning a range of masses and metallicities, reproduce key Milky Way properties such as the dust-to-metal mass ratio, depletion factors, grain size distribution, and extinction curve shape. Crucially, these simulations predict a metallicity-dependent transition in dust growth: in lower-metallicity galaxies, the dust-to-metal mass ratio decreases and extinction curves become steeper in the UV with a weaker 2175 Å bump, reminiscent of the Magellanic Clouds. This trend is attributed to the more efficient gas-phase accretion on silicate grains compared to carbonaceous grains (see Sect. 5.2 in Dubois et al. (2024) for further details of this mechanism) and the preservation of small silicate particles due to low coagulation rates. In higher-metallicity environments, stellar ejecta dominate dust production, resulting in Milky Way-like extinction properties. Together, these observational, laboratory and theoretical studies reveal that silicate dust undergoes significant evolution: from formation in stellar outflows and processing in protostellar environments to extensive reprocessing in the diffuse ISM and galactic-scale feedback in starburst and AGN-driven systems.

### Carbonaceous Grains

Carbonaceous material in the ISM is composed of a wide range of C-bearing molecules, including both crystalline and amorphous carbon, polycyclic aromatic hydrocarbons (PAHs), silicon carbide (SiC), and fullerenes (Dartois 2019). For the purposes of this review, we will focus on the carbonaceous material that forms dust grains in the ISM. As with silicate dust grains, carbonaceous dust grains can be divided into two categories: crystalline and amorphous. The allotropes of carbon are well known, thus the observations of nano-diamond and graphite grains are to be expected. Nano-diamonds were first observed in space in 1999 by Guillois et al. (1999), where they were observed in protostellar discs due to the characteristic C–H stretching modes at 3.43 μm and 3.53 μm, and are made up of $sp^{3}$-hybridised carbon. However, while nano-diamonds are also expected to be abundant in the diffuse ISM, they have yet to be detected in such an environment (Jones and Ysard 2022).

Graphite grains have been indirectly observed in the diffuse ISM through UV extinction (Stecher and Donn 1965), as well as the identification of preserved graphite grains in meteorites (Clayton and Nittler 2004). These grains are made up of $sp^{2}$-hybridised carbon in either monocrystalline or polycrystalline forms, and are indicated by the 2175 Å feature (Draine 2003). Graphene has also potentially been observed in the ISM (García-Hernández et al. 2011, 2012). It is thought that graphene can be produced in the ISM through graphite fragmentation from collisions (Chen et al. 2017).

Amorphous carbon grains can consist of a mixture of $sp^{2}$ and $sp^{3}$-hybridised carbon, which are weakly detected at 3.4 μm (Williams 2005). These grains may also be hydrogenated and are commonly observed in the ISM, serving as significant sources of ISM carbon (Dartois and Muñoz-Caro 2007; Herrero et al. 2022), contributing between 20% and 25% of the total carbon abundance (Draine 2003). It is also worth briefly mentioning grains composed of SiC; these dust grains should be abundant in the diffuse ISM based on the estimated injection rates of carbon-rich stars (Nanni et al. 2021) and pre-solar grains in primitive meteorites (Bernatowicz et al. 1987). Despite this, the strong 11.3 μm absorption feature of SiC extinction has yet to be observed in the ISM (Draine 2003; Dartois 2019). Computational studies by Chen et al. (2021) have shown that the destruction rate of SiC through oxidation would not exceed the expected injection rate. Thus, the lack of SiC in the ISM remains a mystery, and is the topic of current debate, with hypotheses including its serviceability in the atmospheres of carbon-rich stars, overestimates in the injection rate of dust, or underestimates in the size of SiC dust grains. However, SiC_2_ was recently detected in the ISM for the first time by Massalkhi et al. (2023). It is believed that gaseous SiC_2_ is a precursor to SiC in the dust envelopes of carbon-rich AGB stars based on observations of SiC_2_ abundances. As the envelope density increases, the abundance of SiC_2_ decreases, suggesting the accretion of SiC_2_ into dust grains, a process that becomes more efficient at higher densities (Massalkhi et al. 2018).

The vibrational signatures of PAHs have been observed in numerous astronomical environments, ranging from H II regions (Peeters et al. 2002) and planetary nebulae (Smith and McLean 2008; Guzman-Ramirez et al. 2014) to the diffuse ISM and circumstellar shells (Tielens 2008). Moreover, these IR features are not confined to the Milky Way; they have also been detected in various extragalactic sources (Maragkoudakis et al. 2018; Rigopoulou et al. 2021), including starburst galaxies (Brandl et al. 2006; Canelo et al. 2021) and AGNs (Monfredini et al. 2019; García-Bernete et al. 2021; Martins-Franco and Menéndez-Delmestre 2021). In recent years, experimental and theoretical efforts have increasingly focused on super-hydrogenated PAHs (see e.g. Sandford et al. 2013; Cazaux et al. 2016; Yang et al. 2020; Marciniak et al. 2021; Tang et al. 2022), where additional hydrogen atoms are attached to peripheral carbon sites. It has been proposed that, in sufficiently large PAHs, the loss of an extra hydrogen atom can provide an efficient relaxation channel for excess internal energy, thus mitigating carbon backbone fragmentation (Reitsma et al. 2014). At the same time, these super-hydrogenated species may serve as catalysts for H_2_ formation (Rauls and Hornekær 2008; Thrower et al. 2011; Jensen et al. 2019; Schneiker et al. 2022) and hydrogenation reactions (Ferullo et al. 2019). However, several studies indicate that this protective effect is not universal: in smaller PAHs such as pyrene (C_16_H_10_), extensive hydrogenation can instead increase the likelihood of carbon framework fragmentation during collisions or photo-induced dissociation (Gatchell et al. 2015; Wolf et al. 2016; Diedhiou et al. 2020; Stockett et al. 2021). Another possible protective factor is that super-hydrogenated PAHs may be more transparent to higher-energy photons, including X-rays, due to diminished C–C $\pi $ resonance features (Quitián-Lara et al. 2018). Taken together, these findings suggest that the stabilising role of hydrogenation is conditional, depending strongly on PAH size, charge state, and the prevailing environmental conditions.

### Grain Structure and Morphology

As discussed previously, the morphology of dust grains is primarily amorphous, with the majority of dust in the ISM being amorphous silicates and hydrogenated amorphous carbonaceous grains. Crystalline grains also exist in the diffuse ISM in the form of graphite, graphene grains, nano-diamonds, and olivine and pyroxene grains.

Grain size can be inferred from measurements of the extinction and scattering of starlight, with observations of polarisation able to define an upper limit of 500 nm and lower limit of 5 Å (Siebenmorgen et al. 2014). Dust grain sizes typically range from nanometres up to a few micrometres in diameter at their largest. These size distributions are often described by a power law $\mathrm{d}\:n(r)/\mathrm{d}\:r \propto r^{-q}$, where $n(r)$ is the number density of grains of a given radius $r$, and $q$ is an exponent typically determined from polarisation observations (Mathis et al. 1977). The value of the exponent $q$ is generally linked to the region of the ISM and the grain size, with values in the range of $q=3.3-3.6$ generally accepted (though $q=3.5$ is most commonly used) (Mathis et al. 1977; Casuso and Beckman 2010; Siebenmorgen et al. 2014; Weingartner and Draine 2001; Draine 2003). This power law distribution estimates grain sizes between 50 Å and 0.25 μm (Mathis et al. 1977; Weingartner and Draine 2001; Siebenmorgen et al. 2014)

Models often assume dust grains to be spherical or elongated cylinders, as they allow for the simplification of model solutions. However, this is seldom the actual case; dust grains form and grow through repeated collisions, accretion, and condensation, resulting in shapes that are far more irregular and fractal in nature (Min et al. 2007; Draine 2003; Potapov and McCoustra 2021). Indeed, this was confirmed by interstellar dust samples collected by the *Stardust* spacecraft, which revealed rough, uneven surfaces (Westphal et al. 2014). Dust grains are also believed to be highly porous (Ormel et al. 2009), with laboratory experiments on grain formation (Sabri et al. 2013) and cold condensation (Rouillé et al. 2014) estimating porosities of up to 80%. For a comprehensive overview on the topic of dust grain porosity see Potapov et al. (2025).

## Ice in the Interstellar Medium

The ISM is composed of 99% gas and molecular species by mass, and is divided into distinct phases, each characterised by variations in density, temperature, and the ionisation state of the gas, which can range from fully ionised to neutral forms. Ices are present across a wide range of these phases. Neutral material exists in both cold (cold neutral medium, CNM) and warm (warm neutral medium, WNM) regions at temperatures of $\sim 100$ K and $\sim {8000}$ K respectively (Tielens 2005). Stars ionise such gases over a range of temperatures, from $\sim 8000-10^{4}$ K in star forming regions (warm ionised medium, WIM, and HII regions), up to $\sim 10 ^{6}$ K in the atmospheres of stars themselves (McKee and Ostriker 1977; Tielens 2005). Molecular gas resides in giant molecular clouds that can reach 40 pc in size and masses of $4\times 10 ^{5}$ M_⊙_ (Tielens 2005). The cores of these clouds are shielded from high energy external UV radiation by the absorption at the cloud edges (Ferrière 2001), allowing the centres of these clouds to reach very low temperatures of the order of 10 K. At these low temperatures, molecules are able to condense onto the surface of dust grains to form multilayered ice mantles composed of mixtures of molecular species (Muñoz Caro and Escribano 2018). The processes and species that contribute to ice mantle formation will be elaborated in the following sections.

### Ice Species

Ice species in the ISM are typically detected through IR spectroscopy surveys in which absorption of IR radiation from background stars by ice molecules allows for the identification of these species due to their characteristic frequencies. In particular, observations of the vibrational transitions, particularly the stretching and bending mode vibrations in the $3-16$ μm range, allow for the identification of species and specific phases (Boogert et al. 2015). It is also expected that, at the edges of molecular clouds, UV features will be seen (Goebel 1983) due to ionisation by high-energy UV radiation from massive stars. The composition of icy mantles adsorbed to interstellar dust grains is strongly related to the concentration of molecular hydrogen; in H_2_-rich regions, hydrogenated ices such as H_2_O, NH_3_, and CH_4_ form, while in H_2_-poor regions, ices such as CO, O_2_, and N_2_ are more dominant (Whittet 2022). Table 1 summarises the major ice species in terms of their abundance with respect to H_2_O.

**Table 1:** Molecular species detected in ice mantles, their relative abundances compared to H_2_O, and their peak detection positions. References of their specific detections are given in the table footnote

Species	Name	Relative Abundance	$\lambda _{\text{centre}}$ (μm)	Reference^a^
H_2_O	Water	100	3.07	
CO	Carbon Monoxide	0 − 40	4.67	1, 2, 3
CH_3_OH	Methanol	2 − 31	6.85	4, 5, 6, 7
NH_4_^+^	Ammonium	1 − 26	6.755 − 6.943	2, 6, 8
CO_2_	Carbon Dioxide	10 − 25	2.697 − 4.39	9, 10, 11
CH_4_	Methane	0.3 − 23	7.674	10, 12, 13, 14
HCOOH	Formic Acid	1 − 16	7.24	6, 15
NH_3_	Ammonia	1 − 10	2.96 − 9.01	16, 17, 18, 19
OCN^−^	Cyanate	1 − 8	4.598 − 4.617	20, 21, 22
H_2_CO	Formaldehyde	0 − 7	5.83	10, 11, 23
SO_2_	Sulphur Dioxide	0.3 − 1.7	7.63	10, 13,
OCS	Carbonyl Sulphide	0.04 − 0.4	4.90	17, 24, 25

^a^References: 1: Chiar et al. (1994); 2: Boogert and Ehrenfreund (2004); 3: Soifer et al. (1979); 4: Allamandola et al. (1992); 5: Dartois et al. (1999); 6: Boogert et al. (2008); 7: Schutte et al. (1996a); 8: Keane et al. (2001a); 9: Gerakines et al. (1999); 10: Zasowski et al. (2009); 11: Keane et al. (2001b); 12: Lacy et al. (1991); 13: Boogert et al. (1997); 14: Boogert et al. (1996); 15: Schutte et al. (1999); 16: Lacy et al. (1998); 17: Williams et al. (2007); 18: Chiar et al. (2000); 19: Dartois and d’Hendecourt (2001); 20: Tegler et al. (1995); 21: Pontoppidan et al. (2003); 22: Broekhuizen et al. (2005); 23: Schutte et al. (1996b); 24: Palumbo et al. (1997); 25: Geballe et al. (1985).

H_2_O is the most abundant form of ice in the ISM, with the characteristic 3.07 μm band being one of the strongest observed (Van Dishoeck et al. 2013). In the cores of dense molecular clouds, water ices condense onto dust grains, forming a porous amorphous solid water (ASW) (Noble et al. 2024). The phase of this ice can change from amorphous to crystalline over long time scales (Baragiola 2003), or via heating. During heating of the ice, a phase change occurs from high-density ASW to low-density ASW (Jenniskens and Blake 1994), followed by the start of local crystallisation, forming a mixture of amorphous and crystalline phases. This state, often referred to as restrained amorphous ice (RAI), typically occurs at temperatures of ∼70 K (Jenniskens and Blake 1994; Kolesnikov et al. 1999). Further increasing the temperature to between 120 and 140 K results in a cubic crystalline ice (Ic) (Ehrenfreund and Fraser 2003), and finally to an irreversible hexagonal crystalline ice (Ih) after further heating. There also exist many intermediate phases that are pressure- and temperature-dependent, with some unobserved ones likely existing in interstellar environments. Water ice is also the only ice that is present in the atmospheres of evolved stars (Boogert et al. 2015). However, it should be noted that water molecule condensation rates cannot account for the total abundances observed in the ISM, so there must be another mechanism by which water ice mantles can form. Numerous experimental studies (Ioppolo et al. 2008, 2010a; Cuppen et al. 2010; Romanzin et al. 2011) have highlighted various surface hydrogenation reactions in oxygen-rich ice mantles, leading to water ice formation. As such, surface hydrogenation likely accounts for a significant fraction of the H_2_O abundance in ice mantles.

After H_2_O, CO is the second most abundant and second most studied interstellar ice. CO is characterised by an absorption peak at 4.67 μm, and is thought to make up the majority of the outer layers of ice mantles (Boogert et al. 2015; Pontoppidan et al. 2008). CO condenses onto ice grains in a ‘catastrophic’ freeze-out, where CO molecules rapidly condense from the gas phase to the solid phase when molecular clouds reach certain temperatures and densities (Caselli and Ceccarelli 2012). It is in CO-rich outer layers that COMs, molecules having six or more atoms, are thought to form (Chuang et al. 2016; Simons et al. 2020). Experimental studies have demonstrated that phase transitions of CO ice may promote the transport and diffusion of reactive ice species, thus catalysing reactions that form COMs (He et al. 2021). These ice processing mechanisms are elaborated in Sect. 4.

CH_3_OH (methanol) is the most abundant COM present in the ISM. Traced by a prominent absorption line at 6.85 μm, it is primarily formed on grain surfaces through surface hydrogenation reactions of CO (Watanabe and Kouchi 2002; Watanabe et al. 2003, 2004; Whittet et al. 2011), but may also form in the gas-phase (Qasim et al. 2018). Methanol is thought to be a precursor to other COMs (Öberg et al. 2009; Theulé et al. 2013; Fedoseev et al. 2015); similar to CO, the energy released during its formation can lead to partial evaporation. Additionally, some of this energy is released into the ice mantle, causing localised heating and facilitating the movement of reactive species (Caselli and Ceccarelli 2012).

### Ice Mantle Growth

The mechanism of the growth of ice mantles on cold dust grains was first proposed by Lindblad (1935), in which he described the process of H_2_O, NH_3_, and CH_4_ nucleation and growth in the cores of interstellar clouds. In the centre of these clouds, temperatures can be as low as 10 K, sufficiently low that gas-phase species are able to condense onto the surfaces of cold dust grains, with the attachment of these species facilitated through van der Waals interactions. The morphology of the grain surfaces also plays a key role; as discussed in Sect. 2.4, dust grain surfaces are typically highly irregular, thus surfaces will have a number of nucleation sites upon which ice mantles begin to grow.

The formation time of ice mantles can be described through a simple geometric model, as outlined by Whittet (2022):

1 $$t_{m}=\frac{2.5\rho \Delta r}{\xi n{\left(k_{B}T_{g}\right)}^{\frac{1}{2}}},$$

where $\rho $ is the density of the mantle, $\Delta r$ is the mantle thickness, $\xi $ is the sticking coefficient, $n$ is the number density of surrounding gas, $T_{g}$ is the gas temperature, and $k_{B}$ is the Boltzmann constant. This equation can be used to calculate the time taken for a mantle to grow to a given thickness from pure accretion of molecules from the gas phase. Using ice density and thickness parameters from Jones and Williams (1984) and Whittet et al. (1983), and using the Herbst and Leung model (Herbst and Leung 1986) for parameters of a typical molecular cloud, mantle accretion times are estimated to be on the order of billions of years, assuming a sticking coefficient of $\xi =1$ (Whittet 2022); these accretion times are far longer than the estimated $\sim {10}$ Myr lifetimes of molecular clouds. Therefore, ice mantle formation must be dominated by surface reactions rather than only gas-phase accretion. It is also generally agreed that a sticking coefficient of $\xi =1$ is unrealistic, with recent studies suggesting more realistic values of $\xi <0.2$ (Hoose and Möhler 2012; Laffon et al. 2021; Stadler et al. 2024). Such smaller values would result in even longer formation timescales, thus surface reactions become even more important.

Surface reactions, along with the molecular cloud composition, impact the ice species observed in mantles. In young molecular clouds, H is more abundant than H_2_; $\frac{\mathrm{H}}{\mathrm{H}_{2}}>1$. As such, atoms that have accreted onto grain surfaces at this time will interact with H atoms, thus forming hydrogenated ice species such as H_2_O, NH_3_, and CH_4_ (D’Hendecourt et al. 1985; Tielens and Hagen 1982). In older molecular clouds where H has been converted into H_2_ and $\frac{\mathrm{H}}{\mathrm{H}_{2}}<1$, ice species will interact with each other rather than undergoing hydrogenation reactions, so ice species such as CO, CO_2_, O_2_, and N_2_ will dominate. As such, ice mantle growth and composition is highly dependent on the cloud composition and $\frac{\mathrm{H}}{\mathrm{H}_{2}}$ ratio (Allamandola et al. 1999; Whittet 2022). Observations of the vibrational spectra of ices allow for the estimation of this $\frac{\mathrm{H}}{\mathrm{H}_{2}}$ ratio from the net dipole moment of the ice molecules. This leads to a distinct polarity in ices, with hydrogenated ice being ‘polar’, and de-hydrogenated ice being ‘apolar’ (Tielens et al. 1991). Over time, it is expected that mantles will be composed of a mixture of polarities, with an older polar layer rich in hydrogenated species such as H_2_O, topped with one or more apolar layers (Boogert et al. 2015). The abundance of material in these ice layers is often expressed in terms of monolayers, where one monolayer (1 ML) is assumed to be equal to $10 ^{15}$ molecules cm^−2^ (Langmuir 1938).

### Ice Mantle Phases

Observations of ice mantles show that they commonly exist in one of two phases: crystalline and amorphous. Crystalline ices typically exist at higher temperatures, while amorphous phases are observed under low temperature conditions, such as those at the centres of molecular clouds, as well as in regions of high radiation intensity. The origin of these ice phases comes from a number of factors: the ice species itself, the temperature and pressure of the medium, the rate at which atoms and molecules are accreted onto dust grains (Materese et al. 2021; Muñoz Caro and Escribano 2018), and local processing conditions. For instance, in regions of intense radiation, irradiation gradually amorphises the ice. Spectroscopic observations allow for differentiation between crystalline and amorphous phases, with laboratory experiments providing a catalogue of spectra for various ice species with different morphologies. The details of these ice formation experiments will be elaborated in Sect. 5.

**Fig. 2:**
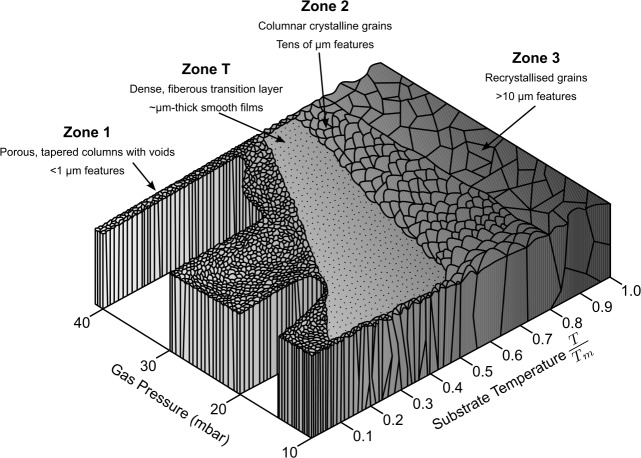
Diagram of the Structural Zone Model showing the characteristic structures and approximate length scales of each zone for films deposited over a range of substrate temperatures and gas pressures. $T_{m}$ denotes the melting point of the deposited material

Laboratory experiments conducted under conditions relevant to astrophysical environments (ultrahigh-vacuum and ∼10 K) by Cartwright et al. (2008, 2010) have revealed that the structure of ice films reflects those predicted by the Structural Zone Model (SZM) at low temperatures. The SZM, developed by Thornton (1974) and shown in Fig. 2, describes how the structure of a deposited film depends on parameters such as substrate temperature and deposition gas pressure. While originally developed to predict the growth of thin ceramic, metal, and semiconductor films for industrial applications (Ohring 2001; Lakhtakia and Messier 2005), the SZM has also been applied to ice deposition under cryogenic conditions. Cartwright et al. (2008, 2010) showed that H_2_O ice films grown on substrates at temperatures between 6 K and 220 K reproduce the morphologies predicted by the SZM. Under low-temperature conditions (6 K), the formation of fractal ice geometries was observed, i.e., high-density amorphous ice. Deposition at laboratory pressures (10 Pa) yielded a ‘cauliflower’ structure, characteristic of Zone 1. Deposition at higher pressures of 113 Pa over shorter time scales yielded the smooth, featureless surface characteristic of the transition structure Zone T. Upon heating these ices to 30 K a transition from high-to-low density amorphous ice was observed. Intermediate temperatures between 20 K and 100 K produced sponge-like and matchstick-like structures characteristic of Zones S and M. At higher temperatures of 220 K, crystalline morphologies resembling those of Zones 2 and 3 were observed. While the deposition pressures and ice film thicknesses considered in these experiments exceed those found in the ISM (where ice mantles typically range from a few nanometers to $\sim 40$ nm), the results still provide valuable qualitative insights into how deposition temperature and environmental parameters influence ice structure and phase transitions. Although mesoscale features require critical thicknesses unattainable under ISM conditions, the underlying mechanisms of structural control remain relevant for understanding processes such as porosity evolution and amorphous–crystalline transitions. Moreover, these studies have direct applicability to planetary science contexts, including comets, icy satellites, and Kuiper Belt objects, where multi-micrometer ice mantles form under similar thermal regimes.

A recent study by Nicolov et al. (2024) observed the nucleation of water ice grains without the need for a dust grain surface to condense upon. In this case, nucleation required the presence of a weakly ionising plasma in which the grains were electrostatically suspended at temperatures between 80 K and 130 K. These grains formed fractal-like elongated structures, with a clear temperature dependence on the grain phase in line with that described previously in Sect. 3.1. These results suggest the possible formation of isolated amorphous water ice grains at the outer edges of protoplanetary discs. While the temperature range considered in this study is not in line with that of dense molecular clouds, the formation mechanism is likely similar, and the formation of such grains would be possible in parallel with the typical ice mantle growth on dust grains in a weakly ionised plasma environment. However, observations of ice formation on the electrodes used in this experiment would indicate that accretion onto dust grains is a more efficient mechanism.

Changes in environmental conditions can lead to changes in ice phase; the process of crystallisation is caused by heating of amorphous ices, or by the continued accretion of gas-phase molecules. These processes impart energy into the mantle, either by direct heating or the transfer of kinetic energy into thermal energy from molecule adsorption (Zhou et al. 1997; Yang et al. 2001), leading to the rearrangement of the ice molecules into a crystalline or polycrystalline phase. There have been numerous studies that have investigated this process both theoretically and experimentally for various ice species, such as H_2_O (May et al. 2012), CO (He et al. 2021), and CO_2_ (Escribano et al. 2013). The point at which crystallisation occurs is dependent on the ice species. Crystalline-to-amorphous transitions are also possible. It is well known that energetic collisions and irradiation processes such as ion and electron irradiation, cosmic ray bombardment, and high-energy UV and x-ray irradiation cause the breakdown of materials. The same is true in ice mantles, with energetic collisional and irradiation processes leading to sputtering and the breakdown of the regular lattice structure. The amorphisation of crystalline water has been observed as a result of ion irradiation by Baratta et al. (1991), Moore and Hudson (1992), Leto et al. (2005), and Mergny and Schmidt (2025), and as a result of Lyman-$\alpha $ photon irradiation by Leto and Baratta (2003).

The impact of these different morphologies has a notable impact on the chemistry that can occur within the ice mantles. This is detailed further in Sect. 4.5.

## Ice Processing

**Fig. 3:**
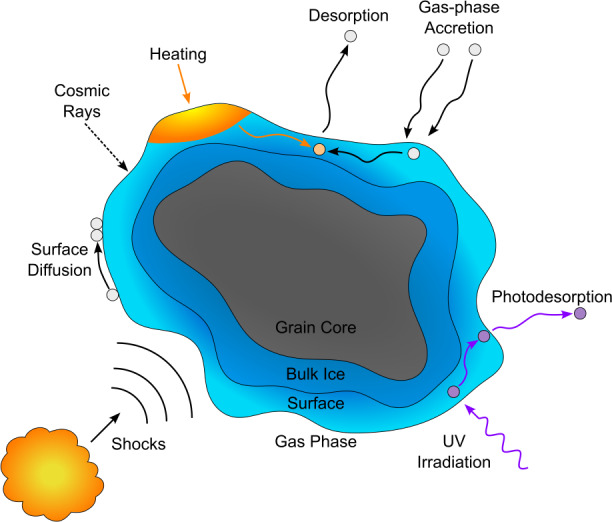
Illustration of the different ice mantle processing methods that may occur in astrophysical environments, including: gas-phase accretion, UV and cosmic ray irradiation, heating, surface processes, shock processing and various desorption processes

Icy dust grains have long been known to act as catalysts for chemical reactions (Gould and Salpeter 1963) and have been studied throughout the latter half of the $20^{\text{th}}$ century; however, it was not until the 1990s that detailed studies were performed. Many of the complex molecules observed throughout the ISM could not have formed through gas-phase reactions alone; thus, processing of ice mantles must lead to this increased complexity. The range of ways in which this processing can occur is complex, covering a wide energy spectrum; the results of this processing are therefore dependent on the processing method and associated energy of the mechanism. Figure 3 illustrates these various processing methods.

The dominant processing mechanism is dependent on the particular time within the dust life cycle. Section 2.1 briefly outlines the life cycle of interstellar dust. While dust resides in molecular clouds, ice mantles begin to form, and processing by irradiation and photochemistry dominates. Shock processing may be present; however, these processing methods are uncommon. When molecular clouds collapse and protostars begin to form, thermal processing from the protostar itself becomes the dominant mechanism. Irradiation via X-rays and shock processing may also occur at this stage. The majority of dust present in the original molecular cloud then goes into the continued formation of stars and planets. New bare dust grains are formed towards the end of the star’s life when it is in the AGB stage, and the cycle repeats.

### Surface Reactions

**Fig. 4:**
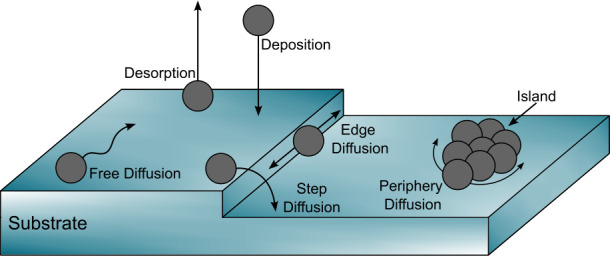
Diagram showing the various interactions of molecules on a surface, including diffusion across the surface, across steps, island formation, and desorption

Surface reactions are the simplest processing method, but also form the foundation for more complex mechanisms such as photochemistry and radiation chemistry. In all cases, whether driven by heating, photon irradiation, or particle bombardment, reactants must encounter one another before they can react to form new molecules. Even intermediate species generated by processes such as photochemistry can only react once they have met another reactant through the meet-and-react pathways defined by surface chemistry. Grain surfaces can catalyse these reactions by providing a substrate on which molecules can diffuse, by offering sites for energy dissipation, and by lowering the activation barriers required for reactions (Potapov et al. 2019). Molecules move across the surface via diffusion, enabling them to cross boundaries or steps, meet other species and interact, or form molecular islands, as illustrated in Fig. 4.

Molecular hydrogen is perhaps the most important molecule in the universe, being the foundation of star formation. Despite this, for many years it was unknown how the abundances of H_2_ observed in the ISM originated, with gas-phase reactions not proving efficient enough (Gould and Salpeter 1963). It is now accepted that the vast majority of astrochemical H_2_ formation is facilitated via surface reactions (Duley and Williams 1984; Gould and Salpeter 1963; Biham et al. 1998). Under high temperature conditions ($>{20}$ K) the presence of the surface acts to catalyse H_2_ production (Roser et al. 2002; Hornekær et al. 2003, 2005) through the Langmuir-Hinshelwood mechanism (Vidali et al. 2006; Le Bourlot et al. 2012; Bron et al. 2014), both by providing a meeting place for H atoms (increasing their encounter probability) and, depending on their chemical composition, by lowering activation barriers for reaction. However, surface reactions rely on the ability of particles to diffuse or tunnel across the grain surface; at low temperatures, diffusion is very slow. This limitation can be counteracted somewhat by accounting for the grain morphology; Hornekær et al. (2005) found that on amorphous surfaces H atoms can accumulate in pores and react to form H_2_, even at low temperatures of 8 K. Thus, grain morphology may play a key role in these surface reactions.

At low temperatures, tunnelling becomes more rapid than diffusion for very light ice species, with the probability increased by the number of surface sites; that is to say that it is increased by surface roughness (Herbst 1995). Recent studies have shown that tunnelling is a key mechanism in the formation of organic molecules (Wang et al. 2023; Marks et al. 2023a).

Surface reactions are not limited to the formation of H_2_, and are key in the formation of a number of more complex species. Some of the most important surface reactions are hydrogenation reactions. Such reactions include the formation of methanol (CH_3_OH) and formaldehyde (H_2_CO) from CO hydrogenation (Linnartz et al. 2015; Watanabe and Kouchi 2002; Watanabe et al. 2003, 2004; Whittet et al. 2011; Potapov et al. 2017), NH_3_ from nitrogen atom hydrogenation (Fedoseev et al. 2014; Le Gal et al. 2014), and methane (CH_4_) from carbon atom hydrogenation (Qasim et al. 2020). Many of these molecules are precursors to COM formation, which may also proceed through surface reactions. Ioppolo et al. (2010b) demonstrated that formic acid (HCOOH) can be formed by OH radical reactions with CO to form HO–CO complexes, which are then hydrogenated to form HCOOH. It was also shown by Ioppolo et al. (2021) that the amino acid glycine (NH_2_CH_2_COOH) is able to form in the first water-rich layers of ice mantles in dark molecular clouds. Glycine is formed from the recombination of NH_2_CH_2_ and HO–CO complexes. NH_2_CH_2_ is formed from HO–CO complex reactions with methylamine NH_2_CH_3_. This process may take place at temperatures as low as 13 K, and may occur prior to the ‘catastrophic’ freeze-out of CO. This glycine may also act as a precursor for the synthesis of further prebiotic molecules via surface reactions (Maté et al. 2015; Oba et al. 2015).

### Photochemistry and Radiation Chemistry

The ISM hosts a very diverse photo-environment, ranging from the protected regions in the cores of dense molecular clouds where very few photons penetrate, to the high-UV environment in the regions around massive stars. Historically, it was assumed that high-energy UV photons would destroy any regions in which chemistry could occur, but we now know that the cores of cold dense molecular clouds provide a protected region in which chemistry can take place. The outer edges of the clouds absorb high-energy UV radiation (up to 13.6 eV) and re-emit them as lower energy secondary UV photons (3-11 eV), which can induce chemistry in ice mantles. High-energy UV radiation or cosmic ray interactions with gas-phase H_2_ molecules in molecular clouds produce a flux of $\sim 10 ^{4}$ photons cm^−2^ s^−1^ lower energy UV photons (Shen et al. 2004). These photons can cause photodissociation and photoionisation, which break down the molecular bonds within the ice mantle and can form reactive species. The recombination of these species, along with interactions with reactive molecules, leads to the formation of new ice species, increasing the complexity of the ice mantle. It is also believed that X-rays may play a key role in planet formation by heating and dispersing material, and inducing turbulence in protoplanetary discs (Feigelson 2010; Joyce et al. 2022; Monsch et al. 2021).

In addition to UV radiation, cosmic rays, X-rays, and electrons can induce radiation chemistry in ice mantles. Shen et al. (2004) concluded that the amount of energy imparted into ice mantles by UV photons is an order of magnitude greater than interactions with cosmic rays, with both UV photons and cosmic ray interactions with the ice mantle producing millions of secondary electrons (Bennett et al. 2005; Mason et al. 2014; Boyer et al. 2016). This cloud of secondary electrons ionises the ice medium along their path and produces free radicals that encourage further chemistry. This can take place through dissociative electron attachment (DEA) (Fabrikant et al. 2017), but is minor when compared to direct ionisation or molecular excitation mechanisms. Experiments conducted by Moore et al. (2007) demonstrated that secondary electrons can efficiently form NH_4_^+^ and H_2_O_2_ in ammonia-water ice films, while Kaiser et al. (2013) formed nine amino acids from electron interactions with CO_2_–NH_3_ ices mixed with hydrocarbons.

The irradiation of ice mantles may also result in the transfer of molecules to the gas phase through desorption or sputtering. This is a fairly important process, as it results in the enrichment of the gas-phase in COMs where they may be detected through sensitive observational techniques such as rotational spectroscopy (COMs cannot be detected in ices using this technique, as rotational motion is restricted in the solid phase). Among the more commonly studied desorption methods is photodesorption, which is a particularly important process under conditions inside molecular clouds where thermal desorption is inefficient (Westley et al. 1995; Paardekooper et al. 2016; Öberg 2016). Sputtering during the bombardment of ices by energetic charged particles can also contribute to the transfer of molecules from the ice to the gas phase, and has been observed and quantified in several laboratory studies using different ions (Brown and Johnson 1986; Baragiola et al. 2003; Famá et al. 2008; Galli et al. 2017, 2018; Ivlev et al. 2023; Dartois et al. 2023) and, to a lesser extent, electrons (Meier and Loeffler 2020; Carmack and Loeffler 2024).

### Thermal Processing

Thermal processing represents the backbone of ice mantle processing; it facilitates the increased diffusion and desorption of reactive species and thus promotes increased molecular complexity. It may be induced by direct heating from nearby star formation, or by other methods of processing such as photo-irradiation and shocks (Caselli and Ceccarelli 2012). Increased diffusion and desorption of both neutral molecules and radicals is facilitated by increasing the kinetic energy of molecular species, resulting in a greater rate of collisions between molecules on the grain surface, thus allowing reactions to take place. In general, it is assumed that only the upper layers of ice are reactive (Hasegawa and Herbst 1993) due to the increased rate of surface diffusion compared to volume (i.e., bulk) diffusion (Gerakines et al. 2000, 2001; Cottin et al. 2003).

There is a diverse range of chemistry that may be induced at low temperatures by thermal processes (Theulé et al. 2013). Ice mixtures of CO_2_ and NH_3_ have garnered particular interest in recent years, as both CO_2_ and NH_3_ are some of the most abundant molecules in the ISM, contain the basic elements needed for biological systems (H, C, N, and O), and may be present in the apolar and polar layers of ice mantles respectively. Several studies have investigated the thermal processing of such mixtures. Bossa et al. (2008) demonstrated that thermal reactions in a 1:1 mixture of CO_2_:NH_3_ ice led to the formation of ammonium carbamate, [NH_4_^+^][NH_2_COO^−^] and neutral carbamic acid NH_2_COOH at temperatures of 80 K. These products only desorbed at temperatures between 230 and 260 K; thus, for a typical molecular cloud temperature of 100 K, it is possible for ammonium carbamate and carbamic acid to reside in the solid phase. Similar results were observed by Lv et al. (2014), who studied the thermal and irradiative processing of CO_2_:NH_3_ ice mixtures. Those authors observed thermal reactions taking place between CO_2_ and NH_3_ starting from temperatures of 80 K. They observed the predominant formation of ammonium carbamate, with peak abundances observed at temperatures of 160 K, before decreasing at 200 K. Carbamic acid formation was also observed from ∼110 K, and persists, only decomposing at higher temperatures in line with results observed by Bossa et al. (2008).

James et al. (2020, 2021) conducted systematic investigations of the stoichiometric mixing ratios of CO_2_:NH_3_ as functions of thermal processing, and of both electron irradiation and thermal processing. In these two studies, seven stoichiometric ratios were considered. In line with the papers discussed previously in this section, ammonium carbamate and carbamic acid are the primary reaction products at temperatures above ∼80 K. However, the stoichiometric ratio of CO_2_ to NH_3_ was found to have a significant effect on the quantity of thermal reaction residues. In CO_2_-rich and equal mixtures, less residue was observed when compared with NH_3_-rich mixtures. This may suggest that NH_3_ enhances the reactivity of the ice. For samples that underwent electron irradiation, a reduced conversion of ammonium carbamate to carbamic acid was observed. The authors suggest that the presence of the irradiation product OCN^−^ acts to stabilise the ammonium carbamate, preventing it from decomposing to carbamic acid upon heating.

Marks et al. (2023b) also demonstrated the formation of ammonium carbamate and carbamic acid from CO_2_ and NH_3_; however, at much lower temperatures than previous studies: 39 and 62 K respectively. Carbamic acid dimers are also observed at temperatures up to 290 K. As these molecules contain amino (−NH_2_), ammonium (NH_4_^+^), carboxylic acid (−COOH), and carboxylate (−COO^−^) groups, they may contribute to the formation of more complex molecules such as amino acids. Due to the high thermal stability of these dimers, those authors posit that they may act as a reservoirs for such amino acids during star and planet formation. Potapov et al. (2019, 2020) further showed the formation of ammonium carbamate and carbamic acid at temperatures between 50 and 80 K. These experiments utilised nanometer-sized amorphous carbon and silicate grains as a substrate upon which to grow the NH_3_ and CO_2_ ices, in addition to a KBr substrate used as a control. They observed a three times greater reaction rate on the grain substrates compared to the KBr control substrate, demonstrating the catalytic role that amorphous grain substrates can play, as outlined previously in Sect. 4.1.

Heating of ice mantles also leads to structural changes, as discussed in Sect. 3.3. When amorphous ice is heated, it undergoes crystallisation, which can lead to impurities becoming trapped in the pores of the amorphous structure. Upon heating, cracks and fissures can form. This causes pores within the ice to collapse, leading to compaction and the rapid desorption of trapped molecules that are more volatile than the bulk ice, in a process known as the *molecular volcano* (Smith et al. 1997). The release of these molecules into the gas phase allows for further gas-phase chemistry to take place using molecules that have already been processed by other means, thus increasing the overall molecular complexity. Multiple experimental studies have observed this molecular volcano phenomenon, in particular with CO (He et al. 2021) and ASW (Collings et al. 2003, 2004; Martín-Doménech et al. 2014). As such, desorption via the molecular volcano may represent a very important desorption method in the case of amorphous ices. Purely thermal reactions involving electronically stable molecules have also been shown to yield numerous routes to COM formation. This mechanism is described in more detail in Sect. 4.6.

### Shocks

Shocks represent an interesting mechanism of ice processing; these events are typically caused by nearby supernovae explosions, protostellar outflows, or cloud-cloud collisions, and as such are a rather rare mechanism of ice processing (Sumpter 2021). Shocks propagating through molecular clouds will compress and heat the cloud components, leading to methods of thermal processing as outlined in the previous section. The nature of the shock affects the heating rate, with J-type shocks exhibiting a more rapid heating, while C-type shocks exhibit a slower, steadier heating rate (Herbst 1995).

Shocks had been proposed as a particular mechanism for the production of CH^+^, an organic compound that is incredibly common in the Orion nebula (Elitzur and Watson 1978) and a key component in carbon chemistry in the ISM. While the formation of CH^+^ is now thought to be driven by high-energy photochemistry (Morris et al. 2016), the formation of similar molecules that require large amounts of energy may well be facilitated, in part, by shock-induced chemistry. Indeed, several studies have shown that shocks may present a key mechanistic route towards the formation of nucleotides (Surendra et al. 2021; Singh et al. 2020; Goldman et al. 2010; Martins et al. 2013).

### Phase Effects

A number of studies have considered how the phase of ice mantles affects the chemistry that occurs as a result of processing. Indeed, Orlando and Sieger (2003), Grieves and Orlando (2005), and Zheng et al. (2007) all considered the irradiation of both crystalline and amorphous D_2_O ices with electron beams, the former two using low energy beams between 5 and 100 eV, while the latter used a 5 keV beam. All three studies obtained similar results, finding increased D_2_, O_2_, and deuterium peroxide (D_2_O_2_) production rates in the amorphous phase. This is attributed to an increased diffusion rate of radiation products of D_2_O through the porous structure of the amorphous ice, preventing recombination back to D_2_O, instead favouring the formation of D_2_, O_2_, and D_2_O_2_. In the crystalline phase these pores are not present, thus reformation of D_2_O post-irradiation becomes more favourable. Zheng et al. (2007) also suggested the contribution of L-type Bjerrum defects in ASW as a possible cause of this increased D_2_O_2_ production. Such defects lead to the favourable recombination of OD radicals to D_2_O_2_ post-irradiation due to favourable orientation of oxygen atoms. For details on the exact mechanism, see the works of Bjerrum (1952) and Zheng et al. (2007).

More recently, Mifsud et al. (2022c) subjected both crystalline and amorphous pure CH_3_OH and N_2_O ices to 2 keV electron irradiation under ultrahigh-vacuum conditions at a temperature of 20 K. In the case of both species, it was observed that the amorphous ices decay more rapidly than their crystalline counterparts, particularly in the case of CH_3_OH, which showed a large relative discrepancy in the decay rates between the two phases; N_2_O showed a smaller difference in the decay rates of its amorphous and crystalline structures. Several factors were identified as potential contributors to this phenomenon, including the need to overcome the lattice energy in crystalline structures and the presence of pores and structural defects in amorphous ices that allow for the increased diffusion of radicals through the ice matrix. The key factor in defining the relative difference in the radiation-induced decay rates of amorphous and crystalline ice phases was suggested to be the strength of the intermolecular interactions within the ice: crystalline CH_3_OH is stabilised by a complex and extensive network of cooperative hydrogen bonds, whereas crystalline N_2_O is stabilised by comparatively weaker dipole interactions, which causes the decay rates of amorphous and crystalline N_2_O to be more similar to one another than in the case of CH_3_OH. Moreover, the increased efficiency of the radiolysis-induced breakdown of amorphous CH_3_OH and N_2_O ices was observed to result in higher abundances of product species compared to the analogous irradiation of the crystalline phases.

In a later study, Mifsud et al. (2022b) presented a systematic investigation of the 2 keV irradiation of all major phases of water ice: ASW, RAI, Ic, and Ih, at temperatures of 20 K. Those authors measured the production of H_2_O_2_ as a result of irradiation, and observed the greatest abundance in the irradiated ASW ice, in line with the results of Orlando and Sieger (2003), Grieves and Orlando (2005), and Zheng et al. (2007). Furthermore, a factor of two decrease in H_2_O_2_ production was observed in the RAI phase, along with a further factor of two reduction on moving from the RAI to the Ic phase. Interestingly, the production rate of H_2_O_2_ in the Ic and Ih phases appeared to be consistent with one another. Therefore, the results of this study are in line with those of previous works of Orlando and Sieger (2003), Grieves and Orlando (2005), Zheng et al. (2007), and Mifsud et al. (2022c), and add further evidence to the idea that amorphous ices decay more rapidly during irradiation compared to crystalline phases. Yet more evidence was found in a later work concerned with the electron irradiation of the amorphous and crystalline phases of solid H_2_S (Mifsud et al. 2022a), which once again demonstrated a more rapid decay of the former. However, it is to be noted that the strength of hydrogen bonds in solid H_2_S is significantly weaker than in, say, H_2_O or CH_3_OH (Bhattacherjee et al. 2013), and thus the more extensive and stronger intermolecular interactions in the crystalline phase cannot be the main factor behind the stability of this phase. Instead, Mifsud et al. (2022a) proposed that the main contributor to the radiolytic stability of crystalline H_2_S compared to its amorphous phase is the lattice energy of the former. Interestingly, SO_2_ ices showed the opposite result, with the crystalline ice undergoing rapid decay compared to the amorphous ice (Mifsud et al. 2022a). Although the exact reason for this result is not fully understood, it has been proposed that the favourable reformation of electronically excited SO_2_ after the dissociation of ground-state molecules in the amorphous phase may contribute to this observation (Souza Bonfim et al. 2017); alternatively, it is also possible that compaction of the ice under the influence of radiation may lead to changes in the band strength constant which would affect the quantitative analysis of the experiment (Mifsud et al. 2022a).

In spite of these experiments, the role of the structural phase of ice mantles seems to have received little research attention despite the key role it appears to play in their chemical productivity. While it appears that the amorphous phase is far more productive than the crystalline phase when subjected to irradiation, a recent study by Suhasaria et al. (2024) showed an increased decay rate in crystalline formamide (NH_2_CHO) compared to its amorphous counterpart. The reason for this observation is not understood and will be the focus of planned future work. Therefore, the influence of the phase of an ice on radiation-induced chemistry seems to still be an open question, and is an area that requires significantly more attention from the community. In Sects. 5 and 6, we discuss the current state of both experiment and modelling to further explore these phenomena.

### Complex Organic Molecules

The formation of COMs in the interstellar medium is still not well understood; however, they are readily observed in star-forming regions (Herbst and Dishoeck 2009; Bacmann et al. 2012). Of the approximately 330 different molecules observed in the ISM, approximately 160 of them are considered to be COMs, as they contain carbon and are comprised of six or more atoms (Endres et al. 2016). Such molecules are the precursors to prebiotic molecules, and therefore, understanding their formation mechanisms is a vital step to understanding the origin of life in the universe. Several COM formation mechanisms have been proposed, with most relying on the processing of ice mantles through the methods previously described, as well as gas-phase reactions.

Numerous laboratory experiments and computational simulations have observed the formation of COMs by various means. Simons et al. (2020) observed the formation of the COMs glycoaldehyde, ethylene glycol, and methyl formate under astrophysical conditions simulating the cores of molecular clouds (8 K) through surface hydrogenation reactions of CO using Monte Carlo simulations. These reactions were facilitated through the surface hydrogenation processes described in Sect. 4.1. These results were later experimentally corroborated by Santos et al. (2022), who additionally demonstrated the formation of methanol via the reaction H_2_CO + CH_3_O → HCO + CH_3_OH. Potapov et al. (2017) demonstrated experimentally that O and H atom bombardment of amorphous carbon grains can lead to the formation of CO. This CO may subsequently undergo hydrogenation to form solid formaldehyde (H_2_CO), one of the reactants in the reaction described by Simons et al. (2020), via hydrogenation.

Given their high energy environments, it is no surprise that there has been a wealth of COMs observed in the hot cores of star-forming regions (Herbst and Dishoeck 2009; Bacmann et al. 2012). The comparatively high temperatures of these regions give rise to a wide range of purely thermal reaction routes to form COMs from electronically stable molecules. For an extensive survey of such reactions, see the work of Theulé et al. (2013) and references therein. Such purely thermal reactions are not subject to some of the limitations of photochemical reactions. The most significant of these is the fact that, in general, photochemistry only occurs in the upper layers of the mantle. In the case of purely thermal processing, reactions can take place in both the surface layers and the bulk region of the mantle; however, as previously outlined, the rate of surface diffusion compared to bulk diffusion is far higher, thus the surface layers are considered more reactive. Mahjoub and Hodyss (2018) conducted laboratory experiments of the purely thermal reactions between the electronically stable molecules methylamine (CH_3_NH_2_) and carbonyl sulphide (OCS) in the context of cometary ice mantles, though as both of these molecules have also been detected in the ISM (Kaifu et al. 1975; Elsila et al. 2009; Suzuki et al. 2016; Drozdovskaya et al. 2018) the results may be analogous to reactions seen in star forming regions. Heating the methylamine-OCS ice mixture from 12.5 K to room temperature resulted in the formation of methylammonium methylthiocarbamate (CH_3_NH_3_^+^CH_3_NHOCS^−^) and thiocarbamic acid (NH_2_CSOH), the latter being formed by the nucleophilic addition reaction between methylamine and OCS, and could be an important step in the formation of thiocarbamate amino acids.

Photochemistry and radiation chemistry present the most diverse routes to COM formation. Throughout the life cycle of interstellar dust, ice mantles become exposed to a range of radiation environments. The mechanisms for COM formation vary with these environments. For example, when ice mantles are in the cores of cold molecular clouds, irradiation from low-energy UV photons as a result of cosmic ray interactions with H_2_ can cause the dissociation of molecules, such as methanol, CH_3_OH, that have formed through the previously outlined mechanism: CH_3_ + OH or CH_2_OH + H. While still under cold cloud conditions, it is possible that these species will remain on the grain surface and will not interact, although recent studies have suggested the potential contribution of so-called ‘non-diffusive’ radical recombinations in the formation of COMs (Jin and Garrod 2020; Garrod et al. 2022). Nevertheless, as nearby protostars form the ice mantle will be heated and these molecules will diffuse more readily, thereby engendering further chemistry. The products of the fragmentation of these molecules can subsequently interact to form more complex molecules such as ethanol, CH_3_CH_2_OH (Garrod and Herbst 2006; Garrod et al. 2008; Öberg et al. 2009), hexamethylenetetramine or HMT, C_6_H_12_N_4_, and polyoxymethylene or POM, (CH_2_O)_*n*_ (Bernstein et al. 1995), to name just a few. HMT is a molecule of particular interest, as when HMT undergoes hydrolysis under acidic conditions, it has been shown to form amino acids (Wolman et al. 1971).

The detection of complex prebiotic molecules such as amino acids in the ISM has continued to elude observational astronomers, although glycolamide (a precursor to glycine) has been detected (Rivilla et al. 2023). Conversely, in experimental studies of ice mantles, amino acids were first detected in 2002 (Bernstein et al. 2002b; Muñoz Caro et al. 2002). In these experiments, amino acids were formed from ice mixtures containing water and methanol, such as H_2_O:CH_3_OH:NH_3_:CO:CO_2_ and H_2_O:CH_3_OH:NH_3_:HCN. Both experiments observed the production of different amino acids through VUV irradiation between 12 K and 15 K, before heating. It has been shown through quantum chemical models that the main mechanism for amino acid formation is the reaction between radicals after photolysis (Woon 2002). Glycine has also been shown to form from ice mantle sublimation in which molecules are repeatedly transferred to and from the gas-phase (Rawlings et al. 2014), as well as from non-energetic radical and atom addition reactions (Ioppolo et al. 2021).

## Experimental Methods

The first attempt to recreate the ice mantles thought to form in the ISM was conducted by Hagen, Greenberg, and Allamandola in the 1970s (Hagen et al. 1979), and the procedure has remained relatively unchanged since then. In order to replicate the true conditions in the ISM, ultrahigh-vacuum and low temperatures are required. Ice is grown on a cold substrate through background or direct deposition, and the composition can be measured through in situ spectroscopic techniques. The ice may then be processed through various means, including irradiation using UV lamps or ion and electron beams. These setups are not able to perfectly replicate the true conditions of the ISM, with the ability to replicate the microgravity environment of space being very difficult; but most significantly, the growth rate of the ice films cannot be accurately replicated. This section will outline the general design of these experiments and highlight some key features and shortcomings.

### Experimental Setup

Replicating the conditions of the ISM requires very low pressure and low temperatures, as well as different radiation environments, depending on the region of the ISM that is of interest. To achieve high ($10 ^{-3}$ to $10 ^{-8}$ mbar) and ultrahigh-vacuum ($10 ^{-9}$ to $10 ^{-12}$ mbar) conditions, vacuum chambers are used with turbomolecular and ion pumps. Baking may also be used to remove adsorbates on the vacuum chamber walls. In order to be able to control the growth of the ice, the vacuum must be carefully managed; for thick ice films, high vacuum conditions are sufficient. To grow very thin ices consisting of only a few monolayers, ultrahigh-vacuum conditions are required to minimise gas-phase contamination, which could alter the structure and thickness of the grown film.

**Fig. 5:**
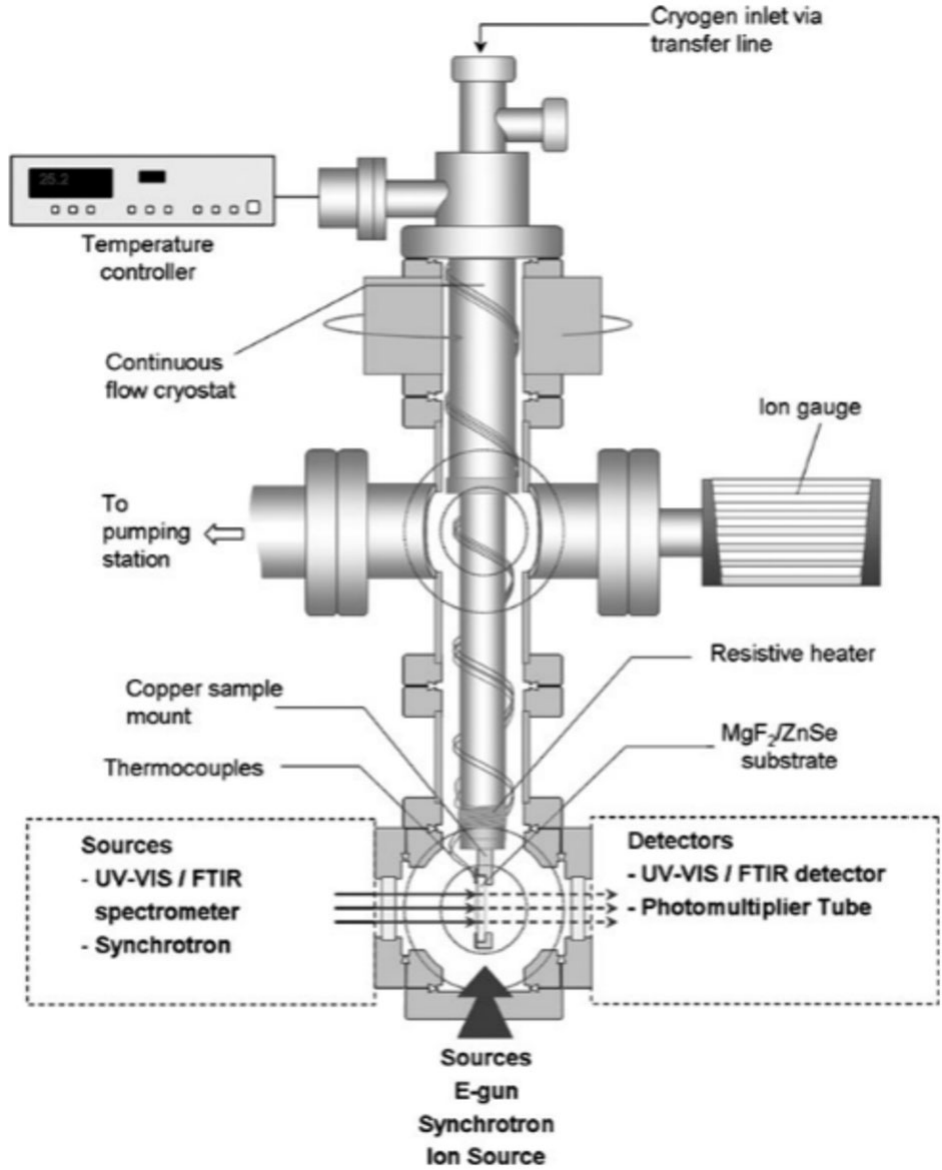
Schematic diagram of a typical astrochemical ice experiment. This particular setup was used in the study of Mason et al. (2006). Image reproduced with permission from the work of Mason et al. (2006)

Ice temperature is typically controlled through the use of a closed-cycle helium expansion cryostat, which can cool the substrate down to $\sim 10$ K. To simulate ice close to protostars, the temperature can be increased. Ice processing is facilitated through irradiation using UV lamps, synchrotron radiation, or electron and ion sources; thus, chambers used for these experiments may need to be located at large-scale central facilities. Figure 5 shows a schematic diagram of such a setup, used for photo-processing experiments of various ice species by Mason et al. (2006).

### Ice Deposition and Substrates

Ice films are experimentally grown through vapour deposition, in which a gas or mixture of gases is introduced into the vacuum chamber. These are then deposited onto the cold substrate either by being injected directly onto the substrate or through background deposition, in which gas mixtures are injected into the chamber and allowed to adsorb onto the cold substrate. The mixtures of gases can be controlled to obtain the desired stoichiometry of ice species, or a single gas can be used to obtain a neat ice species. It is possible to construct layered ices through the subsequent deposition of different species. The rate of deposition will affect the final phase of the ice (Materese et al. 2021; Muñoz Caro and Escribano 2018); however, typical ice experiments last only a few hours with relatively fast deposition rates, astrophysically speaking; in the ISM, ice mantles are expected to grow over very long timescales, on the order of hundreds of thousands of years. The ice thickness will also be dependent on deposition rate, and therefore, the pressure of the injected gas mixture.

The substrate for film growth is normally chosen based on the analysis techniques used. For IR spectroscopy analysis, substrates that are IR transparent such as ZnSe or KBr (Herczku et al. 2021), are used, while for UV experiments, UV-transparent substrates such as CaF_2_ or MgF_2_ may be used (Mason et al. 2006). Metal substrates such as gold may be used where reflection-absorption spectroscopy is desired (Öberg et al. 2007). These choices of substrates, however, are clearly not analogous to dust grains in the ISM. Experiments that use these substrates are typically interested in the surface layers of the ice, rather than the substrate-ice interface. To replicate dust grain surfaces, graphite or silicate substrates may be used (Vidali et al. 2006; Noble et al. 2012; Suhasaria et al. 2015).

### Processing Methods

As outlined in Sect. 4, ice mantles can be processed via various means. Irradiation of ice films is one of the most studied of these processing techniques, allowing for photochemistry and irradiation chemistry mechanisms to be probed. Most photochemistry experiments are concerned with UV light sources, with the fluxes and energies dependent on the region of the ISM of interest. Most experiments consider UV photon energies of approximately 11 eV (approximately the energy of Lyman-$\alpha $ photons), due to the increased radical production compared to ion production that this energy provides, allowing for the specific reaction mechanisms to be elucidated. Higher energy UV and X-rays can be produced using synchrotron facilities, which also allow for an increased level of control over the energy and flux of the photons (Öberg 2016).

To investigate the radiolysis of ice mantles, electron guns and ion sources may be employed. Accelerators, including ion beam facilities and linear accelerators, can generate a wide range of ions, including protons and heavier ions such as carbon and oxygen, at energies that are relevant to those found in various astrophysical environments (Herczku et al. 2021; Mifsud et al. 2021; Rácz et al. 2024). These include giant planetary magnetospheres, in which ions are commonly present (Cooper et al. 2001), stellar wind environments which are often dominated by protons and electrons (Richardson and Stone 2009), and cosmic rays. Cosmic rays may also be simulated through the use of gamma-ray and neutron sources.

Thermal processing is a comparatively simple technique; one simply varies the temperature of the substrate at the desired rate and observes the effect on the ice. The temperature and heating rates may be selected based on factors such as the conditions in the vicinity of a protostar to analyse the evolution of ices in the circumstellar medium.

### Analysis Methods

Analysis of ice films can be split into two broad categories: in situ analysis, that is, analysis that is conducted directly on the ice film as it is being grown and processed, and ex situ analysis, in which the residues left over post-processing are analysed outside of the vacuum chamber, typically using analysis techniques that cannot be conducted in situ.

In situ analysis typically takes the form of spectroscopy, in particular, IR spectroscopy. The reason for this is that the vast majority of astrophysically relevant ice species are IR-active, and thus can easily be identified from the large libraries of ice spectra that already exist. The Cosmic Ice Laboratory^2^ contains IR spectra and IR optical constants, and the Leiden Ice Database for Astrochemistry^3^ (Rocha et al. 2022) contains a database of IR spectra. One of the major advantages of IR spectroscopy is that it is non-destructive, as it does not change the composition or structure of the ice, and it can be used throughout the process of film formation and processing to analyse the composition of the ice as it evolves. IR spectroscopy can also be used to measure the ice thickness by measuring the band strength of each species; as the ice grows thicker, there is greater attenuation of the IR radiation, which is indicative of greater column densities being deposited. Knowledge of the deposited column density, combined with the ice density, means the ice thickness can be estimated (Öberg 2016). A complementary technique for ice thickness measurement is that of laser interferometry: by analysing the interference pattern between laser light reflected at the ice-substrate and ice-vacuum boundaries, the thickness of the ice mantle can be estimated (Goodman 1978; Hudgins et al. 1993; González Díaz et al. 2022).

IR spectroscopy can be configured in two ways: transmission or reflection. In transmission spectroscopy, the radiation is transmitted through the sample and its intensity is measured. The absorption of radiation at specific wavelengths provides insight into the molecular makeup of the sample. This method is commonly known as transmission absorption IR spectroscopy (TAIRS), and all spectroscopic data obtained from telescope observations is based on this transmission spectroscopy method. In comparison, reflection absorption IR spectroscopy (RAIRS) analyses the intensity of radiation reflected from the sample surface. In the context of analysing ice mantles, RAIRS offers a higher sensitivity over transmission spectroscopy when analysing thin films. Transmission spectroscopy offers better sensitivity when analysing bulk substances. RAIRS, however, is particularly sensitive to the type of substrate used, with rough substrates presenting a particular challenge in which the angle of reflection becomes particularly important. Transmission spectroscopy is often used in the laboratory to conduct quantitative analysis of ice samples, such as determining the concentrations and thicknesses.

However, IR spectroscopy is not without its limitations. Because it probes the vibrational modes of functional groups, it is only able to produce unambiguous detections for molecules where these functional groups are present. For more complex species or in ice mixtures, spectral interpretation is complicated by a number of factors; (i) the overlap of absorption bands from multiple species, (ii) shifts in band positions due to mixing (Müller et al. 2021), and (iii) in RAIRS, absorption bands may be split into transverse optical (TO) and longitudinal optical (LO) modes due to the Berreman effect (Baratta and Palumbo 1998; Cooke et al. 2016). This means that even binary mixtures can show shifted bands, making unambiguous identification difficult. These limitations are well documented in the literature, and in cases where ice species are not IR-active, complementary techniques such as Raman or UV-vis spectroscopy are often employed. Chambers are also often equipped with in situ mass spectrometers, in particular, quadruple mass spectrometers (QMS). These are used to monitor the chamber gas composition during the processing phases and during temperature-programmed desorption (TPD), and are not limited by the overlap of spectral features. They may, however, be limited by the overlap of fragmentation patterns, such as in the cases of CO and N_2_, which both possess molecular signals at $m/z=28$. TPD is a vital step in determining the composition of the ice mantle; during TPD the ice substrate is heated such that the ice species desorb. The rate of heating may be varied to replicate various astrophysical conditions. By measuring the gas-phase composition with QMS over time and temperature, it is possible to identify the species that have desorbed. Other, more sophisticated techniques, such as photoionisation reflectron time-of-flight mass spectrometry (Turner and Kaiser 2020), have also been put to use in order to overcome the limitations of IR spectroscopy and QMS. None of these methods, however, currently provides the same broad applicability in the solid phase as IR spectroscopy. For these reasons, IR spectroscopy, despite its limitations, remains the most versatile and widely used tool for the characterisation of astrophysical ice analogues, supported by extensive reference datasets and databases acquired from controlled experiments under astrophysically relevant conditions.

Analysis of the residue left on the substrate after mantle sublimation can be conducted using ex situ techniques such as gas chromatography or high-performance liquid chromatography (HPLC) coupled to mass spectrometry (Attard and Barnes 2004). These residues are often composed of COMs and may also contain prebiotic molecules such as amino acids (Chen et al. 2008; Muñoz Caro et al. 2002; Danger et al. 2013).

### Shortcomings

Experimental techniques are able to replicate a number of the key features of interstellar conditions; however, there are a number of recognised shortcomings. Ice films grown in the laboratory are much larger than the ice films present on dust grains in molecular clouds; typically, the bulk ice samples grown will be on the order of a few micrometres in thickness, while dust grains at their largest are themselves a few micrometres in diameter. As previously described, the thickness of ice mantles that can be grown is linked to the pressure within the chamber: the lower the pressure, the thinner the ice. Current ultra-vacuum techniques are able to achieve pressures of around $10 ^{-12}$ mbar, while the pressure of the ISM can reach as low as $10 ^{-16}$ mbar (Tielens 2005).

The current experimental setups are also not able to replicate the microgravity environment of space, and therefore, the impact of the Earth’s gravitational field on these experiments is unclear. One proposed solution to both of these issues is to use an ultrasonic levitator, in which sound waves are used to trap particles and therefore combat the effects of gravitational fields. This technique would also allow for the use of dust grain analogues of various shapes and sizes, rather than bulk ices on substrates, as is the case with current experiments (Mason et al. 2008). Similar methods using electrostatic levitation in plasmas have been used previously to study the formation of dust and ice grains (Nicolov et al. 2024; Szopa et al. 2006; Shimizu et al. 2010; Ricketts et al. 2011; Chai and Bellan 2015). The use of these plasmas has the additional benefit of allowing the ion chemistry to be modelled. Another proposed solution to the problem of microgravity is to conduct these experiments in the microgravity environment of low Earth orbit, on the *International Space Station* (Ehrenfreund et al. 2003). Microgravity environments have been used extensively in the experimental study of dust agglomeration, making use of drop towers (Blum and Wurm 2001; Blum et al. 2014; Kufner et al. 2011), parabolic flights (Heißelmann et al. 2010; Hill et al. 2015), sounding rockets (Blum et al. 1999a; Brisset et al. 2013, 2016; Molinski et al. 2022), and spacecraft in low earth orbit (Blum et al. 1999b, 2002). For a comprehensive overview of this topic, we direct readers to Wurm and Teiser (2021). Studies of ice formation in microgravity have been conducted in the Kibō module on the *International Space Station* (Yokoyama et al. 2011; Yoshizaki et al. 2012; Furukawa et al. 2017, 2021; Miura and Furukawa 2023), though to our knowledge there have been no studies of ice mantle formation analogous to ISM conditions using any of these methods. This is not unexpected, as placing a full set of experimental apparatus to conduct ice growth experiments as outlined in Fig. 5 on a parabolic flight or in low-earth orbit would be prohibitively complex and expensive. Ehrenfreund et al. (2003) provides a detailed overview of possible ice experiments under various gravitational conditions.

Most current experimental setups do not make use of substrates analogous to dust grains found in the ISM, instead making use of IR-transparent substrates such as ZnSe or KBr. These substrates are appropriate if one is only interested in understanding the composition of ice mantles away from the grain surface, but to understand the role that the grain surface itself plays, accurate substrates are required. There are a number of studies that have considered substrates with structures similar to those of dust grains (usually amorphous carbon): Raut et al. (2012), Fulvio et al. (2012), Sabri et al. (2015), Potapov et al. (2019, 2020), Mifsud et al. (2025). As discussed in Sect. 4.1 and Sect. 4.3 amorphous dust grain surfaces can act to catalyse reactions taking place on the grain surface. This was shown experimentally by Potapov et al. (2019) and later in Potapov et al. (2020), where a factor of three increase in the formation rate of ammonium carbamate was observed.

Furthermore, most experimental studies make use of a one-factor-at-a-time (OFAT) approach (Czitrom 1999), in which a single experimental parameter is varied at a time, such as only varying parameters of a single ice processing mechanism, or conducting irradiation experiments using only photons *or* electrons *or* ions. In reality, all three of these irradiation mechanisms may play a role simultaneously. This may therefore lead to the reporting of results which, while of merit, are highly specific and may not reflect the chemical complexity of the processes taking place in various astrophysical environments. It has been proposed by Mason et al. (2021) that a ‘systems astrochemistry’ approach, in which multiple variables are considered simultaneously rather than individually as has typically been the case with OFAT experiments, may be beneficial. This approach would allow for the use of multiple, simultaneous processing methods akin to astrophysical conditions such as those in the ISM or on the Jovian moons; such an approach is not possible with current experimental setups.

Perhaps the most significant shortcoming of current laboratory experiments is the rate of ice film growth. The phase of the ice films is dependent on the rate at which it is grown (Materese et al. 2021; Muñoz Caro and Escribano 2018), with laboratory growth rates orders of magnitude faster than those in the ISM. This is a very difficult problem to address, as obviously it is not possible to replicate thousands of years of ice formation in the laboratory. Given the impact of ice phase on the chemistry that takes place, as outlined in Sect. 4.5, it is vital to confirm that our current laboratory experiments represent an accurate analogue to ice mantles present in the ISM. However, there is a solution to this problem: with computational simulations, it is possible to simulate these very slow deposition rates. If one were to conduct an experiment in a laboratory and an equivalent experiment using a simulation, any major differences in the results can easily be identified, and the validity of current astrophysical ice experiments can be determined. If one were to identify significant differences between the results of experiments and simulations, it would be of great concern to the astrochemistry community. These simulation methods will be explored in the next section, and a detailed review of the deposition rate problem will be conducted in Sect. 7.1. It should be emphasised, however, that simulations are not a replacement for laboratory experiments. While experimental results are far from meaningless, it is essential to understand how the range of conditions they can replicate fits within the broader context of the ISM.

## Simulation Methods

While experimental studies provide a direct, empirical approach to studying ice growth, simulations offer a number of additional advantages that are not possible with experiments. In some cases, simulations can offer an atomistic level of precision, thus allowing the individual atom-atom interactions that produce particular reaction products to be explored. This level of precision is impossible to obtain from experiments and can provide valuable insight into the ways in which complex molecules are formed. Perhaps the greatest advantage of simulations over experiments is their ability to reproduce conditions that cannot be achieved in the laboratory. This most importantly includes the timescales of interstellar ice mantle formation; using simulations, it is possible to replicate the ice deposition that takes place over hundreds of thousands of years in the ISM.

The process of simulating ice film formation often requires the use of many computational techniques, including different types of simulations for obtaining reaction parameters, modelling ice film growth, and simulating ice processing. In the following sections, we will outline the various techniques that are used and highlight key features and results obtained from these simulations.

### Kinetic Monte Carlo Simulations

Kinetic Monte Carlo (KMC) simulations allow for the precise modelling of individual atoms within an ice film. The motion of particles is modelled as a stochastic process through a random walk, and thus, the motion of the particles is probabilistic in nature. KMC simulations are particularly useful for modelling the diffusion of particles, a process that plays a key role in not only the growth of the ice mantle itself upon molecule accretion, but also the chemistry that takes place, as outlined in previous sections. The work of Cuppen et al. (2013) presents a very thorough review of KMC simulations. In general, KMC simulations are probabilistic: at each time step, there is an associated probability of a particle undergoing a certain event, such as diffusion, dissociation, desorption, or accretion. The probabilities of these events taking place are typically defined through the use of a series of molecular dynamics simulations; the specifics of these will be elaborated in the next section.

The first Monte Carlo simulations designed specifically for astrochemical applications were conducted by Tielens and Hagen (1982), in which they modelled the evolution of the ice mantle from the accretion of single gas-phase molecules. In order to determine the accretion rates, steady-state gas-phase abundances were used; thus, the chemical evolution of the mantle was of more interest than the overall time evolution. Subsequent KMC simulations were conducted by Charnley (1998, 2001) who modelled the chemical evolution using both gas-phase and surface reactions, incorporating the work of Gillespie (1976) in which KMC simulations were used to model coupled chemical reactions.

These studies by Charnley (1998, 2001) considered the overall ice mantle in terms of the abundances of ice species. As such, they may be considered to be macroscopic KMC simulations. In order to consider the specific positions of molecules on the dust grain, microscopic KMC simulations must be used. These are the simulations that make use of random walk dynamics and individual probabilistic events for each particle. To simplify this process, KMC simulations are typically constructed on a grid. This allows for the motion of particles to be considered as moving between these grid spaces, but there is a loss of precision in the exact location of each molecule. Chang et al. (2005) used this method to study the formation of H_2_, finding that inhomogeneous grain surfaces allow for efficient H_2_ formation over a wide temperature range. This is in agreement with experimental studies (Hornekær et al. 2005), in that H_2_ forms in pores within the ice mantle. The difference, however, is that the H_2_ molecules form where H atoms naturally accumulate in areas of the inhomogeneous grain surface itself, where there are crevices or hills that they cannot diffuse over. A follow-up study by Cuppen and Herbst (2005) modelled amorphous carbon and olivine surfaces of varying roughness, and further showed that the presence of surface roughness increased the efficiency of H_2_ formation at temperatures consistent with those of molecular clouds.

Due to its molecular level of precision, KMC simulations can be used to model the growth of ice mantles in great detail, and therefore shed insight into the way in which the ice morphologies observed in both labs and in astronomical surveys are formed. Cuppen and Herbst (2007) studied the formation and phase of water ice mantles on rough carbonaceous grains. In this model, chemistry from surface reactions and photodissociation at various photon energies and fluxes is included. It should be noted that the model is limited to only allowing H and O containing species to react. Results show the formation of water on the grain surface, and not just from gas-phase accretion. Water-forming molecules were deposited over a period of $3 \times 10 ^{5}$ yr, in different cloud environments: diffuse, translucent, and dense. These different environments impact the flux of UV photons on the ice mantles. In the cases with higher gas density, an increase in mantle thickness was observed, along with a greater degree of chemical complexity. In addition, the mantles were amorphous, with large pores observed. In the cases of thick mantles, the lowest monolayers are devoid of H, while the very top layer contains high concentrations of H_2_O. This would agree with the results of previously mentioned studies (Smith et al. 1997) in which radicals formed from irradiation become trapped within the mantle. Upon heating, these molecules may be released through the molecular volcano effect (see Sect. 4.3). These studies highlight the need for accurate determination of the KMC parameters, such as diffusion and desorption probabilities, with small changes leading to very different structures.

Thus, KMC simulations present some of the best ways of modelling ice mantle growth. They allow for an atomic-to-molecular level of detail, while also allowing for very long timescales to be considered, on the order of the timescales of ice mantle formation in the ISM. The key drawback with these simulations is the requirement for accurate parameters defining the events that take place. A number of extensions to classical KMC simulations have been developed (Cuppen et al. 2013), including a unified gas-grain KMC model in which desorption of molecules from the surface to the gas-phase and subsequent gas-phase reactions can be modelled (Chang and Herbst 2012; Vasyunin and Herbst 2012). Off-lattice models may also overcome the restrictions on particle positions in KMC simulations, instead making use of force fields, as is done in molecular dynamics simulations. One notable example of an off-lattice model is that of MIMICK (Model for Interstellar Monte Carlo Ice Chemical Kinetics) developed by Garrod (2013). This model allows for the kinetic modelling of deposition and diffusion processes in 3D. A significant benefit of this off-lattice model is that it is able to account for the effects of the curved grain surface automatically. The functionality of this model has since been extended to include non-thermal diffusion and laboratory conditions (Clements et al. 2018), and the major constituent molecules of interstellar ice (Christianson and Garrod 2021). Off-lattice models may also adopt different variations: Continuum KMC (Zhang and Jansen 2010) models particle diffusion analytically, while Adaptive KMC (Henkelman and Jónsson 2001) adapts between on-lattice and off-lattice simulations to balance accuracy and computational efficiency.

### Molecular Dynamics Simulations

Molecular dynamics (MD) simulations offer a much greater level of detail in the positions of atoms when compared to KMC simulations. Unlike KMC simulations, MD simulations are not based on a grid and therefore allow the precise locations and orientations of atoms to be monitored. The main drawback of this is that MD simulations are limited on timescale, typically only allowing for simulations on the picosecond or nanosecond scales (Cuppen et al. 2013). MD simulations are therefore not particularly useful for the study of ice film growth, but are very useful in the modelling of ice processing. The greater level of detail that MD simulations provide allows for the modelling of processes such as surface reactions, irradiation with UV photons, ions, and electrons, and thermal processing on pre-grown ice films.

In most cases of MD simulations of astrochemical ices, amorphous water ice is used as it is the most common ice species found in the ISM. MD simulations utilise Newtonian mechanics, along with interaction potentials. These potentials define how specific chemical species interact with each other. At each time step of the simulation, the positions, velocities, and other properties of each particle in the system are updated based on Newtonian equations of motion and the interaction potentials. If reactions are being considered, any interactions between particles that would lead to a reaction are calculated (Öberg 2016).

Surface reactions are perhaps the easiest type of ice processing to model using MD. Takahashi et al. (1999) conducted simulations of the formation of molecular hydrogen through surface reactions on amorphous water ice surfaces. They observed the formation of molecular hydrogen via two mechanisms: the Langmuir-Hinshelwood mechanism and the Eley-Rideal mechanism. They also observed a particularly interesting interaction where a near-elastic collision between two H atoms can occur without leading to the formation of H_2_. Another key observation was the accumulation of H atoms in the pores of the amorphous surface, a phenomenon which has also been observed experimentally (Hornekær et al. 2005). In general, these results are in agreement with more recent experimental and computational studies (Roser et al. 2002; Hornekær et al. 2003, 2005; Chang et al. 2005; Vidali et al. 2006; Le Bourlot et al. 2012; Bron et al. 2014).

Simulations of surface reactions with radicals have been modelled by Inostroza et al. (2019), demonstrating the formation of the COM methylene glycol (CH_2_(OH)_2_) from the reaction of CH_3_OH with hydroxyl radicals having energies between 10 eV and 22 eV, in agreement with the experimental results of Shannon et al. (2013). In addition, they demonstrate the favourable formation of methoxy radicals (CH_3_O) compared to hydroxymethyl (CH_2_OH) isomers, in line with detections in the ISM.

MD simulations of photodissociation and photodesorption allow for an atomistic-level investigation of these processes. Arasa et al. (2013) demonstrated CO_2_ formation from the UV photodissociation of CO-H_2_O ice mixtures at molecular cloud temperatures of 10 K. Under these conditions, CO_2_ can form from the photodissociation of H_2_O into H and OH. However, they also found it is more probable for a HOCO complex to form. For efficient CO_2_ formation, the CO molecule needs to be deep within the ice mantle. In addition, it was found that HCO ice can readily form through the interaction between CO and the H photodissociation product, with HCO serving as a key intermediate in the hydrogenation sequence leading to the formation of formaldehyde and methanol (Watanabe and Kouchi 2002; Hiraoka et al. 2002; Fuchs et al. 2009).

The specific process of H_2_O photodissociation has been studied in detail by Andersson et al. (2006) in both crystalline and amorphous phases. In general, there is no difference between photodissociation in these two phases; however, the desorption rates do differ, with H atoms more readily desorbing in amorphous ice, while OH radicals more readily desorb in crystalline ice. As such, these rates would likely have an impact on the CO_2_, HOCO, and HCO formation rates observed by Arasa et al. (2013).

MD simulations have historically been limited by their short-timescale nature, preventing longer-term reactions from being modelled, such as the production of radical species and their subsequent interactions. However, with the recent advent of reactive force fields (Sushko et al. 2016a) it is possible to conduct simulations of irradiation-driven molecular dynamics (IDMD) (Sushko et al. 2016b; Verkhovtsev et al. 2021). This method allows for transformations of complex molecules due to irradiation, and the subsequent reactive transformations of product radicals. To date, no studies have considered this IDMD method in the context of astrophysical ice mantles.

MD simulations are a vital step in developing KMC simulations. In order to determine the probabilities of events taking place in KMC simulations, these must be determined through the use of MD simulations. These include determining the diffusion parameter, for example, by running a series of MD simulations where the molecule of interest is allowed to diffuse across the grain surface. From these models, the diffusion parameter for a specific set of conditions, such as temperature, grain surface, and molecule type, can be determined and provide input into the KMC simulations.

### The Multiscale Approach

**Fig. 6:**
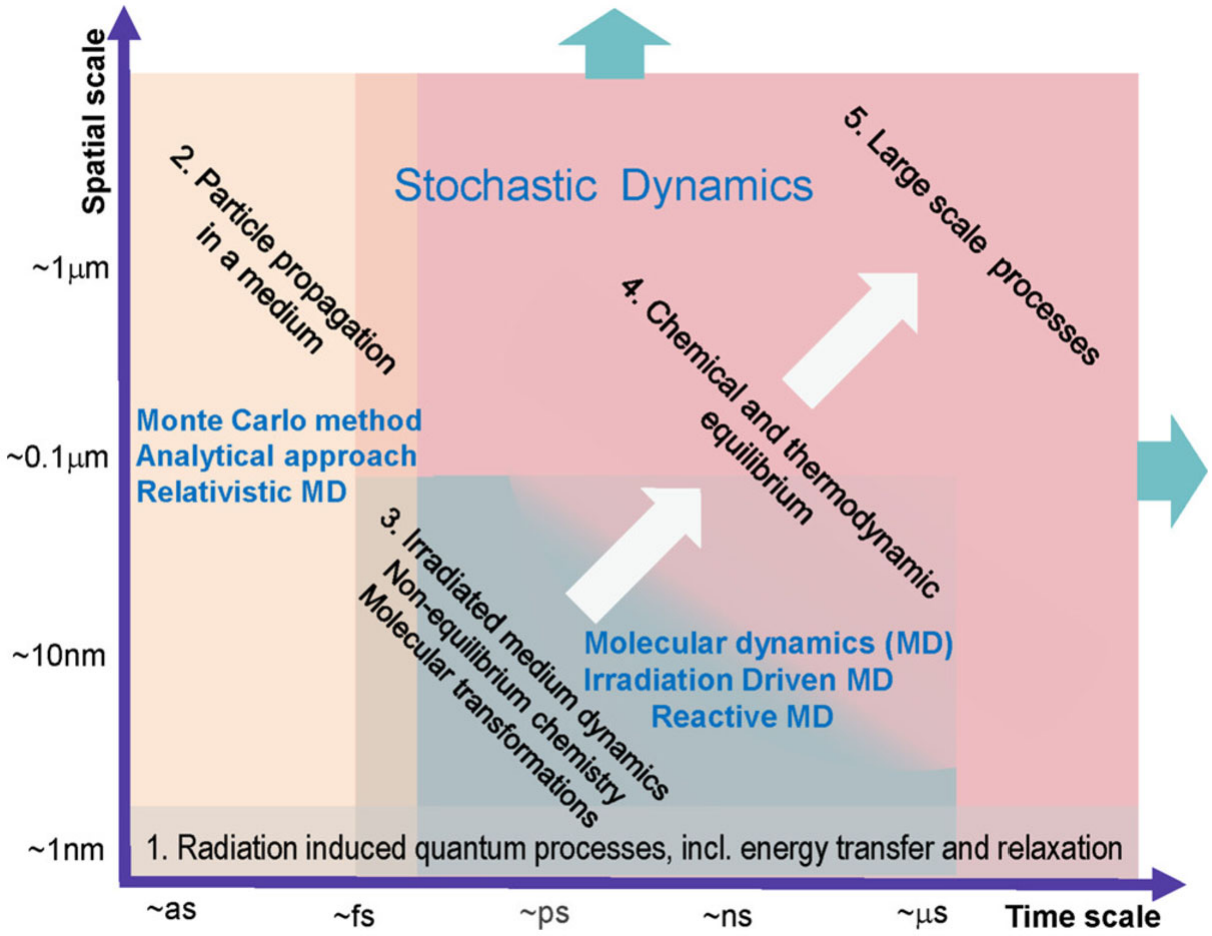
Illustration of the key methodologies used in the multiscale approach to modelling and the spatial and temporal ranges over which they are valid. Different colours correspond to the regions where particular simulation processes are applicable, and they define the boundaries of each method. Image reproduced with permission from the work of Solov’yov et al. (2024)

One of the main drawbacks of current computational approaches to modelling ice mantle growth and processing lies in the requirement for multiple simulation types for modelling different phenomena. It is often the case that these different models are incompatible with each other due to limitations on simulation size and timescales. For example, it is not possible to model accurate photochemistry and irradiation using a KMC simulation due to the lack of spatial resolution, while MD simulations may be able to model these processes but can only do so over time periods that are far too short in comparison to the irradiation timescales in the ISM. A solution to these problems has been proposed by Solov’yov et al. (2024) (among others) in the form of a multiscale approach, in which a wide range of spatial and temporal regimes can be modelled simultaneously. Figure 6 illustrates the scope of such multiscale models, showing the range of spatial and temporal scales over which various methods are valid. Using multiscale methods, it is possible to model individual molecule accretion onto micrometre-sized dust grain analogues, while modelling processes such as diffusion, desorption, and chemical processes by means of KMC probabilities. Multiscale modelling also allows for the coupling of radiation-induced quantum processes, such as the production of secondary electrons and radicals that lead to further chemistry and the formation of more complex species. By direct comparison with experimental results, multiscale simulations can provide a greater insight into almost all of the mechanisms of ice mantle growth, processing, and chemistry that have been discussed throughout this review.

It should be noted that to achieve a full multiscale simulation of interstellar ice, a range of different simulation methods is still required, including KMC (stochastic dynamics), MD, and quantum mechanical simulations. The key difference, however, lies in the available software for conducting such simulations. Historically, different codes developed either commercially or by independent researchers would be used to tackle specific problems, but these codes are often not compatible with each other. There are, therefore, clear advantages to having a single software that can be used to conduct a wide range of multiscale simulations. Currently, one of the most advanced and versatile of such codes are the MBN Explorer (Solov’yov et al. 2012) and MBN Studio (Sushko et al. 2019) software packages, which offer MD simulations, KMC simulations, radiation processes such as IDMD, and biomolecular systems, within a single cohesive package. While certain elements of this software may not be as advanced as some specialised codes for modelling interstellar ices, its diversity and flexibility make it a desirable choice for conducting multiscale simulations, and we expect to see results relevant to the field of astrochemistry in the near future.

### Shortcomings

Computational models are, of course, also not without their shortcomings. These problems range from method-specific issues to areas where the general implementation of the computational methodology can be improved. We will first discuss the method-specific shortcomings, particularly in the cases of MD and KMC simulations. Perhaps the most significant limitation of MD simulations lies in their reliance on accurately parametrised force fields. The predictive power of MD is almost entirely reliant on the suitability of the chosen potential; if the force field does not correctly represent the relevant interatomic and intermolecular interactions, the resulting dynamics will not be physically meaningful. Force-field parameters are typically obtained from quantum chemical calculations, such as density functional theory (DFT), or are fitted to reproduce experimental data. However, while these parameters may be valid for the specific systems and conditions under which they were originally derived, they are not necessarily transferable to other regimes. In astrochemical ice modelling, this can lead to significant errors if, for example, processes such as ionisation, charge transfer, or electronic excitations, common in irradiation chemistry, are absent from the original parametrisation and thus cannot be captured in the simulation. Another major limitation of MD, as discussed before, is the timescales it can access. In practice, MD simulations can only capture processes occurring over nanoseconds or less, making it impossible to directly model slower processes or rare events. This is particularly problematic for astrochemical ices, since low temperatures slow diffusion, restructuring, and reaction rates to timescales beyond the practical reach of MD. The choice of simulation box boundary conditions, such as fixed or periodic boundaries, can introduce artefacts that are inconsistent with real astrophysical environments, potentially biasing structural evolution or energy dissipation. Classical MD simulations also neglect explicit electronic structure and the associated phenomena. Accurate modelling of this usually falls to quantum mechanical approaches such as DFT simulations, which can treat electronic degrees of freedom, but are even more restricted in accessible timescales and system sizes. For further details on MD simulations and their limitations, we direct readers to Berendsen (1999); while an older overview, many of the shortcomings discussed remain relevant today.

A number of the limitations of KMC simulations were already discussed in Sect. 6.1, so we only briefly summarise them here. As with MD, the results of KMC simulations are only as good as the input parameters one provides. These are often derived from experimental data, which is often not available for specific interstellar conditions, in which case MD simulations may be used. This can, however, potentially lead to the compounding of uncertainties if those sources are themselves approximate or not well defined, and should, of course, be validated against experimental data. The on-lattice nature of most KMC implementations can introduce artefacts by constraining motion to discrete sites, reducing spatial precision. Off-lattice models resolve this, but at a significant computational cost, limiting accessible system sizes and timescales. Moreover, the inclusion of many possible stochastic events, such as surface diffusion, edge diffusion, reaction steps, etc., increases both realism and computational expense, meaning that the choice of which processes to include must be made carefully. If key processes are omitted or poorly parametrised, the simulation may fail to capture the true physics or chemistry of the system.

While multiscale modelling offers a powerful way to connect processes across vastly different spatial and temporal scales, it also inherits the limitations of each constituent method and introduces additional challenges associated with coupling them together. One of the major issues comes in bridging between different scales, for example, the determined rates for diffusion, desorption, etc. that are obtained from either MD simulations or experiments may originate from different physical conditions, or have associated uncertainties that are unaccounted for, leading to systemic errors. These may be accounted for by conducting additional validation simulations at the extreme ends of these uncertainties to ensure that parameters have been correctly determined. It is also vital to ensure that the assumptions made are consistent across scales. It can also be quite difficult to validate multiscale results against experiments. While the individual models may be compared to experiments, it is often far harder to benchmark the combined multiscale framework as a whole. Therefore, great care must be taken to accurately document all parameters, their related uncertainties, and assumptions when bridging scales to ensure mistakes are not made.

Finally, we discuss the general issues related to the reproducibility and validity of simulations. As noted in the context of multiscale modelling, benchmarking against experimental or observational data can be difficult. There is no universally adopted standard for validating astrochemistry simulations, and studies often compare against a single, specific case. Ideally, a method should be validated against multiple experimental or observational datasets spanning a range of different physical conditions. In practice, this is rarely feasible, as often models are developed for very specific applications. Nonetheless, the ability for a model to reproduce a single set of independent results lends credence to its predictive power. Reproducibility is further limited by incomplete reporting. Often, fine details such as specific parameters used and their associated uncertainties are omitted, making it more difficult to replicate reported results.

The shortcomings discussed here do not undermine the value of computational models. Rather, they highlight where caution and multiscale thinking are most important. A pragmatic route forward is through modular multiscale workflows: use MD or quantum mechanical methods to determine reaction mechanisms and small-scale parameters, utilise those parameters in KMC or similar frameworks for long-term evolution, and iteratively refine the parameter sets using experimental and/or observational results constraints. As the field of multiscale astrochemical modelling continues to grow, the accuracy of models will no doubt improve. Ensuring that parameters or setups known to correctly reproduce experimental results are readily available is essential. In this regard, the creation of a comprehensive, community-maintained database of validated parameters and techniques would help to greatly streamline the preparation of simulations. With the recent and rapid development of artificial intelligence (AI) and machine learning (ML) models, there has been discussion around their applications within enhancing computational models, such as deriving ML-based force fields for MD simulations (Prašnikar et al. 2024). However, the broader viability of AI in this context remains uncertain, and it remains to be seen what role it may play in the future of astrochemical modelling.

## Open Questions and Applications of Multiscale Modelling in Astrochemistry

As outlined in Sect. 5.5, current experimental methods have a number of shortcomings that lead to some open questions in the field of astrochemistry. Some of these problems can be addressed through the use of computational models, in particular, multiscale models. This topic was introduced in the previous section, and some examples of its applications within astrochemistry were briefly discussed. In this section we discuss in more detail a number of case study topics where multiscale modelling may provide valuable insight were experimental methodologies fall short.

### Deposition Rate

One of the major disadvantages with the current experimental methodology lies in the rate of gas-phase deposition. The rate of gas-phase deposition of a particular species $A$ onto a dust grain may be expressed in units of monolayers per second (ML s^−1^) as (Biham et al. 2001; Cuppen and Herbst 2007):

2 $$R_{\text{dep}}=\frac{v_{\text{A}}n_{\text{A}}}{4\rho },$$

where $n_{\text{A}}$ is the abundance of species A, $\rho $ is the surface site density, and $v_{\text{A}}$ is the average velocity of atoms and molecules in the gas, given by

3 $$v_{\text{A}}=\sqrt{\frac{8k_{B}T_{\text{gas}}}{\pi m_{\text{A}}}},$$

where $T_{\text{gas}}$ is the temperature of the gas, $m_{\text{A}}$ is the atomic/molecular mass of species A, and $k_{B}$ is the Boltzmann constant. If we consider water molecules in a cold dense molecular cloud with a temperature of 10 K, cloud density of $1 \times 10 ^{4}$ cm^−3^, and a site density of $\rho =1 \times 10 ^{15}$ cm^−2^ (Jenniskens et al. 1995), one obtains a deposition rate of $3 \times 10 ^{-14}$ ML s^−1^ or a time of $1 \times 10^{6}$ years to deposit a single monolayer (Cuppen and Herbst 2007). By comparison, typical deposition rates used in experimental setups are on the order of 0.4 nm s^−1^ (Kina et al. 2024; Cazaux et al. 2015), equivalent to approximately 1.3 ML s^−1^ for water molecules, 15 orders of magnitude faster than the rate predicted by Equation (2). This raises the question of whether the results of current experiments accurately reflect the conditions present in the ISM.

Experimental results have shown clear morphological differences in the structures of ice mantles grown at different rates. It is well documented that high deposition rates lead to the formation of crystalline ice structures, while slower deposition results in amorphous morphologies (Kouchi et al. 1994; Gerakines and Hudson 2015b; Kouchi 1990; Gerakines and Hudson 2015a; Gerakines et al. 2023; Hudson et al. 2017, 2015). To form an amorphous ice, molecules adsorbed to the surface do not have enough energy to undergo large-scale diffusion. As such, they do not encounter other molecules and can be thought of as ‘frozen in place’. Kouchi et al. (1994) proposed a criterion for the formation of amorphous ices: the distance that an adsorbed molecule diffuses $D_{\text{s}}$ over the time that it takes to fully cover the surface is smaller than the lattice constant $a$ of the crystalline ice. Therefore, there is a critical flux $F_{\text{c}} = D_{\text{s}}/a^{4}$ for which the deposition flux $F$ of molecules onto the surface must be lower than for an amorphous ice to form, $F< F_{\text{c}}$. The formation of crystalline ice via external processing has been extensively discussed in Sect. 4, however, crystalline ice may also form via non-thermal processes. Molecule adsorption on the grain or mantle surface will impart some small amount of kinetic energy into the system (Zhou et al. 1997; Yang et al. 2001); under high deposition rates, the total kinetic energy imparted into the surface may be enough to induce localised heating, enabling molecular rearrangement into crystalline structures (Gudipati and Castillo-Rogez 2013).

In general, if the timescale over which deposition occurs is longer than that of the time for surface processes to take place, the deposition rate does not play a significant role in the resultant ice phase (Cartwright et al. 2008). This does not take into account the role of ice processing during deposition by heating or radiation processes. To further emphasise the importance of understanding this problem, ice phase has a significant influence on the chemistry that can take place, described in detail in Sect. 4.5. Studies have also shown that the method of deposition has a notable impact on the ice phase. Kimmel et al. (2001a,b) conducted experimental and computational comparisons of ASW ice films grown via background deposition and direct deposition at various angles. By its nature, molecules adsorbed from background deposition are incident on the surface over a random cosine distribution of angles, resulting in a highly porous ice. In comparison, direct deposition allows for precise control over the deposition angles, and therefore, precise control over the porosity. It was found that deposition at angles normal to the surface resulted in films with little porosity, while at angles above $\sim 45$° the porosity increased, with porosities equivalent to background deposition observed at angles between 50 and 55°.

The problem of the influence of slow, long-timescale deposition (similar to that expected to occur in the ISM) on ice mantle phase has received little attention, as these timescales cannot be replicated; however, as outlined in Sect. 6 computational simulations can provide some insight into this issue. A study of particular note is that of Cuppen and Herbst (2007), in which the phase of ice mantles grown under different molecular cloud conditions was modelled by means of KMC simulations. This study was discussed in detail in Sect. 6.1; therefore, we will only highlight the key morphological points here. Ice mantles were grown at various deposition rates in line with conditions in the diffuse, translucent, and dense ISM, over a period of $3 \times 10 ^{5}$ years. In the cases of diffuse and translucent clouds, where the deposition rate is expected to be very low due to the low gas density environment, the mantles are highly porous. These results follow the expected behaviour of slow deposition forming amorphous structures. In comparison, under dense cloud conditions where deposition rates will be higher, a reduced porosity is seen, and ice mantles instead appear to form ‘skyscraper’-like structures. This can be attributed to the relative sticking coefficients between molecules and the grain surface, and between molecules and the ice mantle: if the sticking coefficient between molecules and the mantle is greater than that with the grain surface, molecules will preferentially stick to other molecules in the mantle, thus forming ‘skyscrapers’.

Garrod (2013) conducted a similar study to Cuppen and Herbst (2007), investigating the formation of H_2_O, H_2_, O_2_, and H_2_O_2_ via direct deposition of H and O under different cloud conditions ranging from densities of $2 \times 10 ^{4}$ cm^−3^ to $2 \times 10 ^{7}$ cm^−3^. These simulations made use of the off-lattice KMC model MIMICK, with a particular emphasis on the structure and porosity of the ices. The timescales of these simulations differed for different cloud densities, with the slowest corresponding to $\sim 70{,}000$ years, and the fastest to $\sim 60$ years. The results show an increase in ice mantle porosity with an increase in gas density, implying that higher rates of deposition result in amorphous ice structures, with gas densities analogous to dark interstellar clouds ($2 \times 10 ^{4}$ cm^−3^) resulting in smooth, non-porous structures. The authors therefore conclude that laboratory ices formed through direct deposition rather than background deposition result in much more porous ices than those present in the ISM.

An interesting result comes from the use of an off-lattice model by Garrod (2013): the skyscraper-like structures seen by Cuppen and Herbst (2007) are absent. This is due to the increased precision in particle position offered by off-lattice models. In on-lattice models, particles may only exist at fixed lattice points, while the binding direction depends on the neighbouring particles in the off-lattice model. Therefore, the skyscraper-like structures seen in the results of Cuppen and Herbst (2007) are most likely an artefact of the limitations of on-lattice models.

A follow-up study was conducted by Clements et al. (2018) using a modified version of MIMICK, with the explicit goal of better understanding the role of different deposition parameters on ice mantle structure. In particular, they studied the effect of temperatures between 10 and 120 K, and a range of background deposition at rates consistent with different regions of the ISM and laboratory conditions: dense molecular cloud conditions of 10 molecules cm^−2^ s^−1^ ≡ $10 ^{-14}$ ML s^−1^, protoplanetary disc conditions of $10^{6}$ molecules cm^−2^ s^−1^ ≡ $10 ^{-9}$ ML s^−1^, and laboratory conditions of $10^{13}$ molecules cm^−2^ s^−1^ ≡ $10 ^{-2}$ ML s^−1^ and $10^{16}$ molecules cm^−2^ s^−1^ ≡ 10 ML s^−1^. Deposition rates in line with experiments showed good agreement between the structure and porosity of laboratory-grown ices. However, under protoplanetary disc conditions, ice mantles were seen to be less porous when compared to laboratory-grown ices at low temperatures, while porosities for temperatures between $\sim 30-40$ K aligned better with experimental results. In the case of dark interstellar cloud conditions, there is a clear difference between experimental and simulation results; under low-temperature and low-deposition rate conditions, ice mantles were found to be much less porous. The authors posit that this is a result of the timescales considered: under low-deposition rate conditions, molecules have more time to thermally diffuse across the surface before they are fixed in place by the adsorption of other molecules. By contrast, in experiments, the comparatively high deposition rates mean there is insufficient time for the molecules to rearrange themselves before additional molecules adsorb and fix existing molecules in place. In all deposition rate cases, increasing temperature led to a reduction in porosity. However, there is little difference between the ice structures at 10 K versus 35 K, thus indicating that thermal diffusion is responsible for much of the rearrangement. Therefore, under low-temperature conditions, non-thermal processes become dominant. Such processes may include the energy gained from the adsorption of molecules onto the grain surface, as previously outlined. This study also considered deposition at specific angles ranging from 0 to 75°. In agreement with the results of Kimmel et al. (2001a,b), increased deposition angles lead to an increase in porosity, with angles of $\sim 60$° leading to similar porosities to background deposition. Kimmel et al. (2001a,b) did not consider different deposition rates or temperatures; however, the results of Clements et al. (2018) showed a clear temperature dependence. High deposition temperatures resulted in reduced porosity, likely due to the greater available energy allowing for diffusion and rearrangement. Ices grown at laboratory deposition rates ($10^{13}$ molecules cm^−2^ s^−1^) were much more porous compared to ices grown under dense molecular cloud conditions ($10^{1}$ molecules cm^−2^ s^−1^) for all deposition angles, indicating that regardless of deposition angle, laboratory-grown ice structures differ from those expected to exist in dense molecular clouds.

The experimental study by Notesco et al. (2003) considered the effects of argon gas in pores in water ice mantles using deposition rates between $10^{-1}$ and $10^{-5}$ μm min^−1^, equivalent to between 5.56 and $5.56 \times 10^{-4}$ ML s^−1^. This work was conducted in the context of comet formation; however, the mechanisms are applicable to ice mantle growth in the ISM, with the trapping efficiency of Ar being very similar to that of CO. At high deposition rates ($10^{-1}$ μm min^−1^) and temperatures between 22 and 27 K (analogous to those present in the ISM) there was a large amount of trapped Ar in the ice mantle, higher than the amount of water ice itself. With decreasing deposition rate, the amount of trapped Ar decreases, in line with the results of most other studies. This is attributed to the ability of gases to diffuse out of any pores before they are covered by an additional layer of ice at low deposition rates, while the gases are trapped at higher rates. In this study, the porosity of the ice mantle itself was not analysed, only the total gas that was released during TPD, and thus it is not clear what role the deposition rate plays in the overall ice structure. It is possible that under lower deposition rate conditions, the ice is more crystalline, thus there is nowhere for additional Ar to become trapped. However, in these experiments, the deposition rates are still far higher than those expected in the ISM, and thus the results must be extrapolated to a lower deposition rate, as with all experimental studies. Nevertheless, this is one of the few experimental studies that does consider a range of deposition rates.

The deposition rate problem is perhaps one of the most significant questions in the field of ice astrochemistry, and it is not one that has been particularly well studied. While it is impossible to replicate the required timescales in a laboratory environment, KMC simulations have only been applied sparingly to this issue. The results of such simulations have highlighted deviations from experimental results, with Garrod (2013) and Clements et al. (2018) showing that laboratory-grown ices are much more porous when compared to simulated ices. Further investigation is required to probe the direct effect of deposition time and its coupling with evolving ISM thermal and radiation conditions, since these factors can promote the emergence of both crystalline and amorphous phases. Current KMC simulations are also limited by the lack of accurate interaction parameters, required to provide an accurate representation of a particular environment in which ice mantles can grow.

### Irradiation-Induced Chemistry

As outlined in Sect. 4.2, irradiation represents one of the most diverse routes to increased chemical complexity in the ISM. It encompasses a wide range of energetic processes, including photon irradiation from UV, X-rays, and gamma-rays, as well as particle irradiation from electrons and ions. These interactions lead to reactions in both the surface layers of ice mantles, as well as in the bulk, in part due to secondary electron production. Unfortunately, laboratory experiments suffer from similar limitations to those discussed in the previous section on ice growth: experiments are inherently limited in their timescale. Radiation fluxes in the ISM are many orders of magnitude lower than those achievable in the laboratory, meaning that experimental timescales are typically compressed from $10^{5}$–$10^{6}$ years to hours or days.

MD simulations are particularly valuable for studying short-term irradiation chemistry. Their atomistic resolution allows for a detailed understanding of physical and chemical processes, including sputtering and reaction pathways. Unlike conventional MD, reactive force fields such as ReaxFF (Duin et al. 2001) or IDMD (Sushko et al. 2016b) allow for bond breaking and formation, enabling changes in particle type during a simulation. ReaxFF in particular has been used in the context of astrochemistry in the work of Mainitz et al. (2016), in which they investigated the effects of cosmic-ray ion irradiation on amorphous H_2_O:CO_2_:NH_3_:CH_3_OH ice mixtures. The ice, modelled as an amorphous sphere without a substrate or layered structure, was irradiated with a simulated ion track depositing 18.23 keV into the grain. This produced a shock wave that heated the ice to 5000 K within 1 ps, followed by rapid cooling to 300 K by the end of the simulation. The energy deposition led to the ejection of molecular clusters such as H_2_O and CO_2_ along with their fragments. Within the ice grain, efficient formation of NH_4_^+^ was observed, though it was rare in ejecta. Other abundant products included OH, H_2_, O_2_, H_3_O^+^, H_2_O_2_, CO, H_2_CO, CH_2_OH, and H_3_CO. The atomistic nature of MD simulations allowed the exact formation pathways to be traced, allowing specific reaction mechanisms to be identified. The authors note a number of interesting examples: O_2_ was primarily produced from CO_2_ fragmentation, while H_2_ originated from the fragmentation of H_2_O, NH_3_, and CH_3_OH. H_2_CO formation involved C atoms from both CH_3_OH and CO_2_, while O originated from CO_2_ and H_2_O. Recombination reactions were also observed, with H_2_O formation being most common, followed by NH_3_. This ability to distinguish between primary fragmentation products, secondary recombination products, and their ionic or radical character is a unique strength of reactive MD simulations. The authors also compare their results to experimental studies of swift-heavy ion irradiation of similar ice mixtures. In general, they show agreement in the types of products formed, though they note their lack of N_2_ and O_3_ production in the simulations. They attribute this to the smaller number of reactive species and the short reaction timescales accessible in MD. By comparison, experimental conditions produce a greater number of reactive species due to the higher overall irradiation fluxes.

A similar study was conducted by Schrauwen et al. (2025), in which they performed IR free-electron laser (FEL) irradiation of amorphous H_2_O:CO_2_ (1:4) ice mixtures of various thicknesses with complementary MD simulations to investigate the vibrational energy dissipation and its influence on the ice structure. Experimental results of on-resonance irradiation of the CO_2_ asymmetric stretch at 4.215 μm showed that the dissipation of energy from these vibrational modes into H_2_O may lead to restructuring in the thicker ices, consistent with non-heating. The authors note that such changes may also occur in thinner ices but could remain undetected due to signal-to-noise limitations, and could arise from segregation following CO_2_ desorption or from vibrational energy transfer from CO_2_ to the H_2_O OH stretch. In contrast, irradiation at the CO_2_ bending mode (14.88 μm) yielded much weaker observable effects, likely due to lower photon absorption at this wavelength. By comparison, the results of MD simulations showed that energy from the CO_2_ bending mode dissipates rapidly into intermolecular and H_2_O vibrational modes, effectively heating the H_2_O over short timescales of a few hundred picoseconds. The CO_2_ asymmetric stretch is highly localised by comparison, transferring energy mainly to other asymmetric stretches and only coupling slowly to CO_2_ librations and H_2_O twist modes after 2 ns, possibly contributing to the restructuring observed in experiments. Here, the timescale is particularly important: the FEL experiments, the 1 ns off-time between pulses is shorter than the slow 2 ns dissipation timescale. This means that the system remains partially excited when the next pulse arrives. In the ISM, irradiation will occur on much longer timescales, with the IR photon flux orders of magnitude less than can be provided by the FEL setup. This suggests that in the ISM, the interaction between the CO_2_ asymmetric stretch and the H_2_O vibrational modes may be less significant than observed in experiments. However, preliminary results suggest that the arrival time between pulses is not the main factor. It is therefore clear that more work is required to properly understand this mechanism. Nevertheless, the combined experimental–computational approach is invaluable, as it allows for the connection of macroscopic restructuring in the ice to specific molecular-scale energy transfer pathways. This study highlights the limitations presented by the use of high-flux processing methods such as FELs, where energy is deposited into systems far faster than is possible under interstellar conditions. This raises the question of if such processing techniques are relevant in studies of ISM ice mantles. They may, however, be much more relevant to study impacts where large amounts of energy are deposited over very short timescales, as has been done by the work of Ferus et al. (2019).

Modelling irradiation chemistry is not restricted to MD simulations, however. While MD simulations are able to capture short timescale effects and provide details on reaction mechanisms, they are generally unsuitable for studying processes on timescales longer than a few nanoseconds. To model irradiation on astrophysically relevant timescales ($10^{5}$–$10^{6}$ years), we must turn to either KMC or rate-equation models. In practice, KMC studies mainly focus on ice growth, structure, and very simple reactions, as outlined in the previous section. Rate-equation models, on the other hand, treat the abundances of gas-phase, surface, and bulk-ice species by solving coupled differential equations that describe the net rates of all chemical processes, such as accretion, desorption, photodissociation, and surface reactions. Reaction rates are typically expressed as products of species abundances and reaction rate coefficients, such as thermal activation, tunnelling probabilities, or non-thermal processes such as irradiation effects. For a full overview of how such models work, we direct the reader to Ruaud et al. (2016), which provides a detailed overview of the Nautilus model. In general, these methods are very computationally efficient as they treat species as continuous variables, and are therefore able to model timescales of up to $10^{8}$ years. The drawback of these methods, however, is that they do not model the precise position of particles, and therefore cannot capture structural properties such as porosity at the grain scale. (Cuppen et al. 2013).

Shingledecker and Herbst (2018) presented a general methodology to implement radiation chemistry into rate-equation models, which was later implemented by them into Nautilus in Shingledecker et al. (2018). This study focuses on chemistry occurring in cold molecular cloud cores, where thermally driven processes such as surface diffusion are strongly suppressed. They modelled cosmic-ray irradiation of cold cloud sources, including the Taurus Molecular Cloud 1 (TMC-1), as well as hypothetical clouds with identical conditions but exposed to varying cosmic-ray ionisation rates. The ionisation rates were determined based on laboratory-derived G-values and reaction branching ratios. Their results showed that the non-thermal processes induced by cosmic-ray irradiation can produce substantial abundances of astrochemically relevant molecules such as HOCO, NO_2_, HC_2_O, and the COM HCOOCH_3_. The dominant reaction pathway to the formation of these species is through radiolytic dissociation, indicating that they can be formed even at the low temperatures of cold molecular clouds. The inclusion of radiation chemistry improved the agreement between modelled and observed abundances of HC_2_O in TMC-1. Increasing the cosmic-ray ionisation rate enhanced the abundances of many species at early times of $\lesssim 10^{3}$ years, but at longer timescales of $\sim 10 ^{6}$ years, radiolytic destruction processes became dominant, leading to net decreases in abundance.

A follow-up study by Shingledecker et al. (2019) used the MONACO code (Vasyunin et al. 2017) together with the radiation chemistry formalism developed in Shingledecker and Herbst (2018) to model keV proton irradiation of laboratory ices. Two experimental systems were considered: pure H_2_O ice at temperatures of 16 and 77 K, and pure O_2_ ice at 5 K. Results of the H_2_O irradiation showed that the model reproduced the experimentally measured abundances of H_2_O_2_ at both temperatures (Gomis et al. 2004). In the O_2_ irradiation case, the simulations also matched the observed formation of O and O_3_ (Baragiola et al. 1999). The latter case is particularly relevant in the context of multiscale modelling: the same system had previously been modelled by Shingledecker et al. (2017) using a more computationally expensive KMC approach, yet the simpler rate-equation model in Shingledecker et al. (2019) reproduced the bulk product yields equally well. This suggests that for studies focused solely on radiolytic product abundances, rate-equation models may serve as an efficient alternative to more detailed stochastic simulations. However, as previously noted, rate-equation models cannot capture structural effects that KMC and similar models can resolve.

This section is by no means a comprehensive overview of computational methods of radiation chemistry. Many other reviews, such as Cuppen et al. (2024), go into far more detail on laboratory and computational advances in ice chemistry, including irradiation pathways, ice morphology, and how microscale processes inform astrochemical evolution across environments. Nonetheless, the case studies presented here illustrate the multiscale nature of irradiation chemistry: from molecular-scale energy deposition and reaction pathways, to the incorporation of radiolytic effects into grain-surface networks, and how models can now bridge the gap between ultrafast irradiation physics and chemical evolution. Looking ahead, further refinement of this multiscale methodology is essential; challenges still include (i) extending models to capture bulk-diffusion and ice restructuring effects, (ii) refining and obtaining more appropriate reaction rates, (iii) validating rate-equation models against the more complex KMC simulations, and (iv) integrating observational data so that laboratory-informed mechanisms can be tested in astrophysical contexts. With JWST already revealing the chemistry of the cold ISM in unprecedented detail, refining these multiscale models is more timely than ever.

### Dust Formation and Growth

The mechanics of dust grain formation and growth in interstellar environments are still not well understood. Once dust grains reach a certain size, they begin to interact gravitationally and start the gravitational stages of planet formation. However, the processes that lead grains to grow to these gravitationally relevant sizes still remain an open question. In Sect. 5.5 we discussed a number of experimental methodologies that have been used to study dust agglomeration in microgravity environments. However, the precise processes by which nanometer-sized grains coagulate to form the larger grains typically associated with ice mantles are still not well understood.^4^ Understanding these processes is vital not only for understanding the grain structure of dust normally associated with ice mantles, but also for understanding planet formation.^4^ In this section, we will discuss some of the computational approaches to investigating dust grain formation and growth under interstellar conditions and highlight the role that multiscale computational models may play in future studies.

The problem of dust coagulation and agglomeration is indeed a multiscale one: on the smallest scales at the earliest stages of dust formation, models must consider molecule-molecule interactions. As grains grow, so does the spatial scale that needs to be modelled: as dust grains grow to sizes where ice mantles become relevant, they are nano to micrometers in size, and even larger still when considering the earliest stages of planet formation. To obtain a full understanding of the mechanics of dust grain growth, a wide range of computational techniques, from DFT to hydrodynamic simulations, is required.

We start our discussion at the smallest scales: the earliest stages of dust grain formation occur between nanocluster grains that have originated from the outflows of AGB stars or supernovae. Rimola and Bromley (2021) conducted DFT simulations with periodic boundary conditions of proto-silicate Mg_6_Si_3_O_13_ nanocluster aggregation to form bulk silicate aggregates. In order to mimic the aggregation process, the periodic unit cell of the nanocluster was systematically reduced, bringing nanoclusters from neighbouring periodic images close enough to begin to interact. They found that by sufficiently decreasing the periodic unit cell, the structures evolved from nanoporous aggregates to dense amorphous silicates, with the aggregation primarily triggered by the formation of Mg–O bonds. To compare the structure of these grains to those observed in the ISM, simulated IR spectra were calculated from the vibrational frequencies of the systems. For isolated nanoclusters, the IR spectra were identical to that of observed Mg_6_Si_3_O_12_ . In the case of the bulk silicate aggregates formed from the aggregation process, the calculated IR spectra are in good agreement with observational spectra of silicate grains from Henning (2010).

MD simulations can provide valuable insight into the larger-scale processes of dust grain growth when grains reach nanometer and micrometer sizes. Rosandi et al. (2024) provides a detailed overview of the methodologies needed to perform such MD simulations. A number of studies have conducted MD simulations of dust grain collision and coalescence in astrophysical contexts. Kempf et al. (1999) conducted such MD simulations of dust grain growth driven by Brownian motion. These simulations model the diffusion dynamics of micrometer-sized particles under typical protoplanetary disc conditions (100 K and $2 \times 10^{-2}$ Pa). The formed aggregates were found to be highly porous and structurally diverse, forming fluffy, fractal-like clusters rather than compact grains, consistent with the samples collected by the *Stardust* spacecraft (Westphal et al. 2014). The friction times, the strength of the interactions between the dust grain and surrounding gas, increase only weakly with aggregate mass, leading to growth rates that are far too slow to account for the rapid formation of planetesimals observed in astrophysical environments. These results suggest that Brownian motion alone is insufficient to drive efficient dust growth and must be supplemented by other mechanisms, such as turbulence or differential settling, to enable the formation of larger bodies within the lifetime of a protoplanetary disc.

Collisions also play a key role in dust agglomeration. For the purposes of MD simulations, the mechanics and results of these collisions can effectively be boiled down to a number of *recipes*, which were pioneered by Dominik and Tielens (1997), and provide an overview of the expected sticking, restructuring, fragmentation, or compaction of the collided grains as a function of the impact energy. This set of recipes was further expanded through MD simulations by Paszun and Dominik (2009), in which erosion and further fragmentation routes were identified.

Other works have considered the role of ice mantles on dust grain growth. These ice mantles should impact the sticking coefficient of the grains, enhancing grain formation. Bossion et al. (2024) conducted MD simulations of amorphous carbon grains at temperatures between 50 K and 2250 K to calculate the sticking coefficients of gas phase H, H_2_, C, O, and CO. These results are applicable to the study of ice mantle growth, but also for dust grain growth. Results under high temperature conditions associated with stellar atmospheres where species such as C and H are present, showed that H atoms desorb more rapidly from grain surfaces as compared to C atoms. Therefore, during the early grain growth phases, sticking is dominated by other C atoms compared to H atoms. This leads to rapid depletion of C, whereupon H accretion becomes more favourable, and may lead to the formation of PAHs. A similar study was conducted by Alfaridzi et al. (2023), in which they conducted MD simulations of porous silicate dust grain collisions. In this work, they compared the sticking rates between base SiO_2_ grains and porous grains with enclosed water ice pockets and a water ice mantle. In collisions between porous grains with no water ice pockets or ice mantles, sticking did occur and the grains did not bounce off each other over a range of collision velocities between 150 and 1200 m s^−1^. By comparison, bare grains with no pores showed sticking up to velocities of 900 m s^−1^, and grains with no pores and the water ice mantle showed decreased sticking efficiency up to 200 m s^−1^. The presence of pores filled with ice pockets results in an intermediate sticking efficiency, with sticking occurring up to collision velocities of 600 m s^−1^. This suggests that the presence of ice mantles on dust grains inhibits the sticking and therefore coalescence and grain growth process; similar results were obtained in the authors’ previous work (Nietiadi et al. 2020) when only considering grains coated with an ice mantle. The authors posit that the reason for this decrease in sticking efficiency when ice mantles are present is due to the ice mantle preventing contact between the grain surfaces, which, in the case of SiO_2_, have a large number of dangling bonds. These results are in contrast to previous studies (Wang et al. 2005) that have shown ice mantles lead to increased sticking due to electric charge interactions.

We now turn our attention to the largest-scale simulations of dust grain growth and evolution: hydrodynamic (HD) simulations. These are a type of simulation that deals with spatial and temporal scales that are far larger than those considered for ice mantle growth. Such HD are the cornerstone of computational astrophysics, modelling fluid dynamics, and are used over a wide range of applications from star formation and galactic evolution, to modelling the large-scale structure of the universe and planet formation. It is the latter that is of most interest here.

Hirashita and Yan (2009) developed a magnetohydrodynamic (MHD) model to investigate how interstellar turbulence drives dust grain evolution via shattering and coagulation across different phases of the ISM. They considered the evolution of both silicate and graphite grains with starting sizes between $10 ^{-3}$ μm and 0.25 μm. Simulation timescales ranged from 1 to 100 Myr, depending on the ISM phase, with shorter durations ($\sim 1-5$ Myr) for ionized media and longer durations ($\sim 10-50$ Myr) for neutral and dense phases. The results of these models show that large grains $\gtrsim 10 ^{-2}$ μm are efficiently shattered in the warm ionized medium, where gyro-resonance accelerates grains to high velocities, while in dense clouds the low relative velocities allow small grains $\lesssim 10 ^{-2}$ μm to coagulate efficiently. The results suggest that repeated cycling between diffuse and dense phases leads to a balance that regulates the grain size distribution. Furthermore, shattering in the warm neutral medium may constrain the maximum grain size to around 0.25 μm, while coagulation in dense environments can influence the lower size cutoff.

These HD simulations are also a very good example of the multiscale nature of dust growth simulations. HD simulations either rely on parameters from MD or other dynamical simulations of dust collisions to determine the rates of collision, fragmentation, etc., or they may be directly integrated into the HD models. This is the case in Beitia-Antero and Castro (2021), which presents a dust collision model integrated into a 2D MHD simulation that allows dust coagulation to be modelled in molecular cloud envelopes. They model dust growth of both charged silicate and carbonaceous grains starting from grain sizes between 50 Å and 0.25 μm over periods of around 3000 years. Over this period, silicate grains show the most growth, reaching radii of $\sim {0.34}$ μm on average, while graphite grains show much slower growth and only reach radii of around 0.25 μm. They also observed that, in general, there was a greater overall probability of grain fragmentation than coagulation for both grain types.

Bate (2022) investigated the coagulation and growth of dust grains during the earliest stages of star formation: during the gravitational collapse of static molecular cloud cores and the formation of the first hydrostatic core (FHC). These models used 3D smoothed particle hydrodynamics (SPH) simulations to model the evolution of grain sizes, incorporating factors such as Brownian motion and turbulence. Starting from graphite grains with sizes between 5 nm and 0.25 nm, the models show that prior to FHC formation, grain growth is minimal; however, after the FHC forms, growth becomes rapid, producing grains larger than 100 μm. They also considered rotating molecular clouds, which accelerates grain growth to up to 0.5 mm due to overdensities and turbulence within the formed spiral arms of the FHC disc. These findings suggest that significant grain growth can occur before stellar core formation, especially in rotating systems, and highlight the importance of including early-stage coagulation in models of dust evolution and planet formation.

This overview has only touched on a small fraction of the available literature covering dust grain formation and growth. Comprehensive discussions can be found in books and reviews such as Testi et al. (2014), Marel and Pinilla (2024), Birnstiel (2024), and Potapov et al. (2025) which examine the topic from laboratory, observational, and theoretical perspectives across the stages of star and planet formation. The examples presented here underscore the diversity and complexity of the grain growth problem, as well as the number of open questions that remain. This is fundamentally a multiscale process, with molecular-scale simulations—such as MD or cluster dynamics—providing sticking coefficients, fragmentation thresholds, and other microphysical parameters that feed into large-scale HD and population-synthesis models of dust evolution. Moving forward, challenges include bridging the gap between collision-scale physics and disc-scale evolution, incorporating realistic grain morphologies and compositions, and constraining models with spatially resolved ALMA and JWST observations. As these links between scales are strengthened, dust growth models will become increasingly predictive, helping to reveal the pathways from interstellar grains to planetary systems.

## Conclusions and Outlook

The field of astrochemistry has grown rapidly in recent years. With the advent of JWST, the rate at which new molecular species are being discovered in the ISM has dramatically increased (McClure et al. 2023). Understanding how these species form and the roles they play is the foundation of numerous areas of research, from star and planet formation to the genesis of life itself. In this review, we have discussed the key mechanisms that underpin the formation of these complex molecules. Dust grains formed at the ends of the lives of massive stars play a crucial role in the formation of complex molecules, acting as a surface on which complex chemistry can take place. These dust grains have rough, inhomogeneous surfaces, providing nucleation sites where accreted gas-phase molecules can more readily meet and react. This helps to overcome the very slow rates of diffusion in cold molecular clouds, where molecules would not be able to come in contact with one another. It is these grain-surface interactions that are perhaps the most important, being responsible for the vast majority of H_2_ production, without which stars would not be able to form. Ice processing by thermal heating, photoionisation, and irradiation play a key role in the emergence of the chemical complexity in these mantles. Thermal heating allows for increased diffusion rates, overcoming the diffusion limitation of cold dust grains. The diverse photo-environment of the ISM leads to photoionisation and photochemistry, promoting increases in molecular complexity, along with interactions with cosmic rays, X-rays, and electrons. These mechanisms also play a key role in liberating molecules from the surface phase into the gas phase by means of desorption, photodesorption, ice sputtering, and the molecular volcano effect, thus allowing for further gas-phase chemistry to occur.

We have also highlighted the key experimental and computational methods that are utilised to study the growth of ice mantles in the ISM. The ability to accurately reproduce the conditions found in the ISM is essential to understanding the mechanisms by which these ice mantles form and evolve, and this is only possible by using a combination of experimental and computational techniques. Experimental methods provide a direct approach to exploring ice mantle formation, allowing for precise measurement of both the physical and chemical properties of the ice and the results of processing. However, they struggle to replicate the true conditions of the ISM, in particular, there are limitations in the achievable vacuum conditions, the lack of a microgravity environment, substrates that are not an accurate mimic of real dust grains, and most importantly, the rate at which ice mantles grow, being orders of magnitude faster under laboratory conditions than in the ISM. Computational studies are able to overcome a number of these issues, as they are able to simulate ice mantle growth over periods of millions of years and under a range of ISM conditions through the use of KMC simulations. MD simulations may be used to accurately model the processing of ice mantles including thermal heating and irradiation, providing atomistic-level insight into their impact on ice mantle structure and the chemistry that can take place, with IDMD allowing for the accurate modelling of specific radiation-driven processes. In addition, by utilising multiscale models, simulations over a wide range of spatial and temporal scales can be conducted.

Computational simulations rely on having an accurate set of parameters, be those inter-atomic potentials in the case of MD simulations, or KMC probability parameters in the case of KMC simulations. As such, for models to be accurate, these parameters must also be accurate. Such parameters are often available in databases, such as a database of binding energies between ice molecules and grain surfaces needed to model desorption (Cuppen et al. 2017); however, these databases are often not tailored to specific astrochemistry cases. Therefore, as models of interstellar ices become more complex, a specific database of inter-atomic potentials or KMC parameters relevant to astrochemistry is needed.

Taking all of these experimental and computational methodologies into account, we can anticipate that the coming years will be incredibly productive in the field of solid-phase ice astrochemistry. Experiments are becoming ever more accurate; however, they are now starting to be complemented by ever more detailed computational models. The recent interest around multiscale models presents a number of exciting avenues for research, in particular, understanding the role of the immense timescales of these processes on the chemical evolution of the ISM. It is only by combining both experimental and computational techniques that we can begin to fully understand the physical and chemical processes that take place in the ISM, and perhaps how this leads to the emergence of life.

## Data Availability

Not Applicable.

## Footnotes

1 See the Cologne Database for Molecular Spectroscopy.

2 NASA Cosmic Ice Laboratory.

3 Leiden Ice Database for Astrochemistry.

4 This is a topic of the COST Action CA22133 The birth of solar systems (PLANETS).
